# A quantitative comparison of the deleteriousness of missense and nonsense mutations using the structurally resolved human protein interactome

**DOI:** 10.1002/pro.70155

**Published:** 2025-05-19

**Authors:** Ting‐Yi Su, Yu Xia

**Affiliations:** ^1^ Graduate Program in Quantitative Life Sciences McGill University Montréal Québec Canada; ^2^ Department of Bioengineering McGill University Montréal Québec Canada

**Keywords:** deleteriousness of mutations, functional consequences, genotype‐to‐phenotype relationships, human genetic variants, human protein interactome, interactome disruptions, Mendelian diseases, missense and nonsense mutations, protein stability, structural systems biology

## Abstract

The complex genotype‐to‐phenotype relationships in Mendelian diseases can be elucidated by mutation‐induced disturbances to the networks of molecular interactions (interactomes) in human cells. Missense and nonsense mutations cause distinct perturbations within the human protein interactome, leading to functional and phenotypic effects with varying degrees of severity. Here, we structurally resolve the human protein interactome at atomic‐level resolutions and perform structural and thermodynamic calculations to assess the biophysical implications of these mutations. We focus on a specific type of missense mutation, known as “quasi‐null” mutations, which destabilize proteins and cause similar functional consequences (node removal) to nonsense mutations. We propose a “fold difference” quantification of deleteriousness, which measures the ratio between the fractions of node‐removal mutations in datasets of Mendelian disease‐causing and non‐pathogenic mutations. We estimate the fold differences of node‐removal mutations to range from 3 (for quasi‐null mutations with folding ΔΔG ≥2 kcal/mol) to 20 (for nonsense mutations). We observe a strong positive correlation between biophysical destabilization and phenotypic deleteriousness, demonstrating that the deleteriousness of quasi‐null mutations spans a continuous spectrum, with nonsense mutations at the extreme (highly deleterious) end. Our findings substantiate the disparity in phenotypic severity between missense and nonsense mutations and suggest that mutation‐induced protein destabilization is indicative of the phenotypic outcomes of missense mutations. Our analyses of node‐removal mutations allow for the potential identification of proteins whose removal or destabilization lead to harmful phenotypes, enabling the development of targeted therapeutic approaches, and enhancing comprehension of the intricate mechanisms governing genotype‐to‐phenotype relationships in clinically relevant diseases.

## 1 INTRODUCTION

**FIGURE 1:**
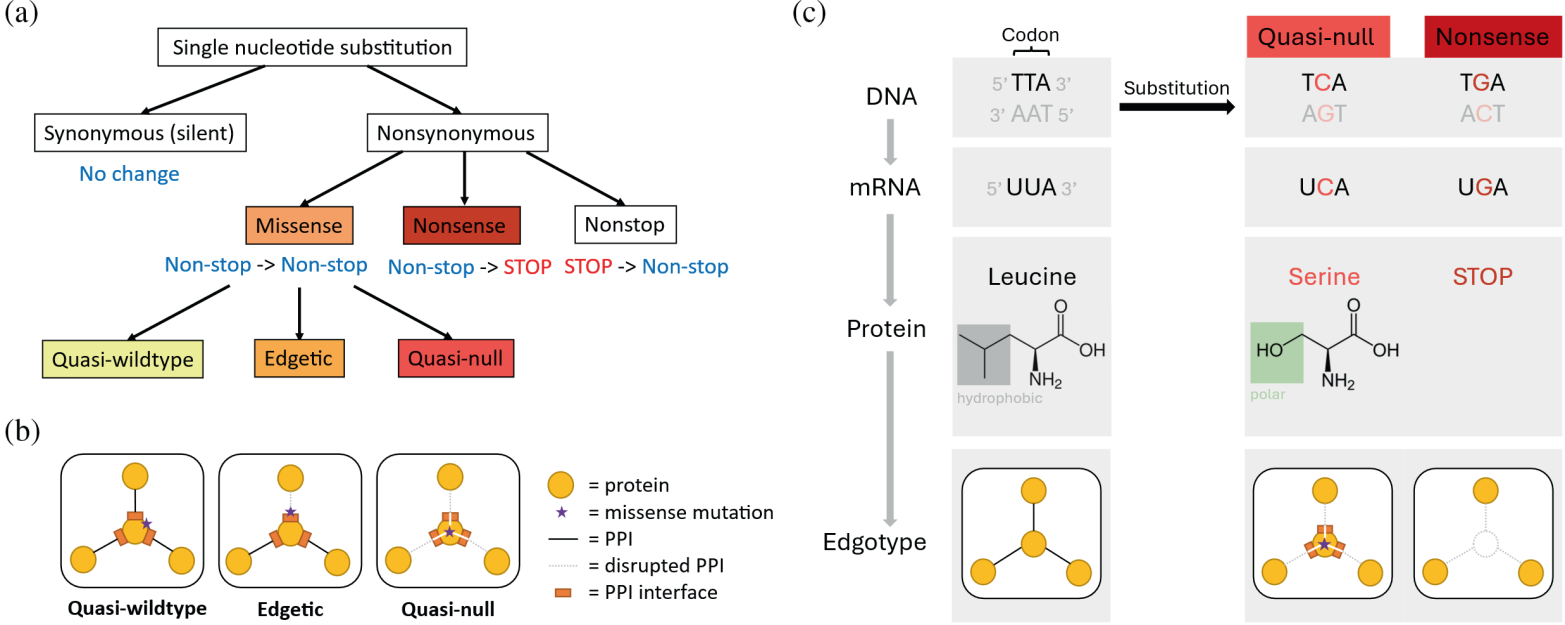
Depiction of two types of nonsynonymous single nucleotide substitutions: missense and nonsense mutations. (a) Single nucleotide substitution tree illustrating the relationship between missense (orange) and nonsense (dark red) mutations. A missense mutation is a nonsynonymous substitution in which a non‐stop codon is changed into another, resulting in a change in amino acid in the protein. A missense mutation can be categorized into three different “edgotype” classes (quasi‐wildtype, edgetic, and quasi‐null) based on its edge‐specific effects on the protein interactome. A nonsense mutation is a nonsynonymous substitution in which a non‐stop codon corresponding to an amino acid is changed to a stop codon. (b) Diagram portraying the interactome perturbations of quasi‐wildtype (left), edgetic (middle), and quasi‐null (right) mutations. (c) A comparison of quasi‐null (bright red) and nonsense (dark red) mutations at the DNA, mRNA, protein, and edgotype levels.

Even simple Mendelian diseases, arising from germline mutations in single genes, have complex genotype‐to‐phenotype relationships (Vidal et al., 2011). This complexity has been attributed to perturbations in the molecular interaction networks (interactomes) in human cells, as changes in these circuitries can lead to large differences in cellular behavior and contribute to the development of disease phenotypes (Ideker & Sharan, 2008; Zanzoni et al., 2009). Nonsynonymous single nucleotide substitutions, such as missense and nonsense mutations, have been shown to cause disruptions in the human protein interactome, the network of all protein–protein interactions (PPIs) (Sahni et al., 2013; Zhong et al., 2009). A missense mutation changes a non‐stop codon into another corresponding to a different amino acid, whereas a nonsense mutation converts a non‐stop codon into a premature termination codon (Figure 1a). These distinct genetic changes result in specific perturbations in the protein interactome and lead to phenotypic effects with varying degrees of severity. Thus, investigating the functional implications of interactome disruptions provides a direct assessment of the mutations' phenotypic consequences and enhances our understanding of the intricate mechanisms underlying genotype‐to‐phenotype relationships in clinically important single‐gene diseases.

Although mutations causing premature termination codons (nonsense mutations) are generally considered more deleterious than missense mutations, it is unclear how functionally comparable nonsense and missense mutations are. Combined Annotation‐Dependent Depletion (CADD) has been introduced for estimating the deleteriousness of human genetic variants, including single nucleotide variants and small insertions and deletions (Kircher et al., 2014). CADD integrates diverse genome annotations, such as conservation scores (GERP [Cooper et al., 2005], phastCons [Siepel et al., 2005], phyloP [Pollard et al., 2010]), regulatory information, transcription factor (TF) binding information, and protein‐level scores (Grantham [Grantham, 1974], SIFT [Ng & Henikoff, 2003], PolyPhen‐2 [Adzhubei et al., 2010]) into a support vector machine to calculate C‐scores that measure the deleteriousness of mutations. CADD estimates median C‐scores for all genome‐wide nonsense and missense variants to be 37 and 15, respectively. Although CADD provides a precise quantitative assessment of the deleteriousness of mutations, its annotations cannot explicitly differentiate between missense and nonsense alleles, and the functional comparability of missense and nonsense mutations remains unclear. For example, GERP, phastCons, and phyloP scores assess evolutionary conservation at the nucleotide level, and thus cannot distinguish nucleotide substitutions that result in missense or nonsense mutations in the resulting protein. Furthermore, SIFT and PolyPhen‐2 scores predict the effect of amino acid substitutions on protein function, making them applicable only to missense mutations. Hence, we propose that investigating the specific perturbations caused by missense and nonsense mutations in the human protein interactome provides a comprehensive comparison of the mutations' functional consequences, thereby enhancing our understanding of their deleterious effects.

To effectively evaluate the interactome disruptions induced by missense and nonsense mutations, missense mutations must first be differentiated based on their “edgotypes” (edge‐specific perturbations) (Sahni et al., 2013). Edgotypes of missense mutations range from no detectable change in PPIs (“quasi‐wildtype”), to a specific loss of one or more PPIs (“edgetic”), to a complete loss of all PPIs (“quasi‐null”) associated with the protein carrying the mutation (Figure 1b). Structural systems biology approaches that employ high‐resolution human protein structural interactomes (SIs), comprising three‐dimensional (3D) structures for PPIs, have recently been introduced for predicting edgotypes (Cui et al., 2018; Ghadie & Xia, 2019, 2021, 2022; Kiel & Serrano, 2014; Marrero et al., 2024; Mosca et al., 2015; Xiong et al., 2024). Such computational approaches have yielded results that are broadly consistent with experimental procedures (Chavez et al., 2015; Sahni et al., 2013). Among the three edgotypes of missense mutations, quasi‐null mutations are the most analogous to nonsense mutations as they both result in protein (node) deletions in the protein interactome. A quasi‐null mutation produces a complete protein initially, but it affects the protein's folding stability and eventually leads to a loss of the protein (node) and its PPIs (edges) (Figure 1c). A nonsense mutation produces a transcript containing a premature termination codon that is typically degraded by the cell's nonsense‐mediated mRNA decay (NMD) system before it can be translated into a protein product (Lykke‐Andersen & Jensen, 2015; McIntosh et al., 1993). If a nonsense mRNA escapes NMD, it results in a truncated protein that is generally non‐functional (Dyle et al., 2020). Consequently, a nonsense mutation immediately appears as a node deletion in the interactome (Figure 1c). Edgotyping missense mutations allows quasi‐null mutations, which share similar functional consequences to nonsense mutations, to be highlighted and systematically compared to nonsense mutations.

The mechanisms through which quasi‐null and nonsense mutations lead to node deletions in the interactome differ substantially; a quasi‐null mutation destabilizes the protein product, while a nonsense mutation does not typically produce a protein product. It is unclear how such distinct mechanisms influence the functional deleteriousness of quasi‐null and nonsense mutations. The severity of a quasi‐null mutation can be quantified by the change in stability of the protein upon mutation. Variability in the efficiency of NMD has been shown to affect the pathology of genetic diseases (Doma & Parker, 2007; He & Jacobson, 2015; Holbrook et al., 2004; Khajavi et al., 2006; Miller & Pearce, 2014), suggesting that NMD efficiency reflects the harmfulness of a nonsense mutation. However, it is difficult to quantify NMD efficiency for a nonsense mutation, as the efficiency differs from cell‐to‐cell and a significant portion of nonsense transcripts escape NMD for unknown reasons (Lappalainen et al., 2013; Linde et al., 2007; Lindeboom et al., 2016; Rivas et al., 2015; Sato & Singer, 2021). Thus, a straightforward calculation applicable to both quasi‐null and nonsense mutations is crucial for analyzing their functional impacts on the interactome and for assessing and comparing their overall deleterious effects in the population.

In this work, we employed structural systems biology approaches to construct high‐quality SIs at atomic‐level resolutions for characterizing the edgotypes of missense mutations, allowing for a systematic comparison of missense and nonsense mutations. We obtained Mendelian disease‐causing and non‐pathogenic missense and nonsense mutations and mapped them onto our SIs. These mutations originate from patient samples, providing clinical relevance and reflecting genetic diversity in the human population. Leveraging 3D protein structures in our SIs, we performed structural and thermodynamic analyses to edgotype and assess the biophysical implications of these mutations. Such analyses enabled the identification of a specific type of missense mutation, known as “quasi‐null” mutations, that exhibit comparable functional outcomes (node removal) to nonsense mutations. We calculated the effects of quasi‐null mutations on protein stability, defined here as folding ΔΔG (folding ΔG_mutant_ − folding ΔG_wildtype_). We developed a quantification termed a “fold difference” for estimating the deleteriousness of mutations. Our fold difference calculation conceptually resembles a fold change measure of differential gene expression between disease and wildtype phenotypes (Love et al., 2014). We defined the fold difference for nonsense mutations as the ratio between the fractions of nonsense mutations in datasets of Mendelian disease‐causing and non‐pathogenic nonsynonymous mutations. Similarly, we defined the fold difference of quasi‐null mutations as the ratio between the fractions of quasi‐null mutations in datasets of Mendelian‐disease causing and non‐pathogenic missense mutations. We estimate the fold differences of node‐removal mutations to range from 3 (for quasi‐null mutations with folding ΔΔG ≥2 kcal/mol) to 20 (for nonsense mutations). We observe a positive linear relationship between fold difference and protein instability (folding ΔΔG), indicating that the deleteriousness of nonsense mutations is comparable to that of severe quasi‐null mutations (with folding ΔΔG >40 kcal/mol). These findings suggest that the gap in deleteriousness between quasi‐null and nonsense mutations can be reduced by increasing the folding ΔΔG cutoff (≥2 kcal/mol) in determining quasi‐null mutations. Overall, the wide range in folding ΔΔG caused by these mutations demonstrates that the deleteriousness of quasi‐null mutations spans a continuous spectrum, with nonsense mutations at the extreme (highly deleterious) end. We further tested for potential factors confounding our fold difference calculations and observed that fold differences are not confounded by the extent of the structural models' coverages of the proteins in our SIs, validating the applicability of our structural systems biology approaches. By calculating fold differences between datasets of Mendelian disease‐causing and non‐pathogenic mutations, we provide a simple and universal measure for assessing and comparing the functional and phenotypic effects of quasi‐null and nonsense mutations in clinically important single‐gene diseases. Overall, our work provides a better understanding of the intricate mechanisms underlying genotype‐to‐phenotype relationships in human diseases, allowing for the potential identification of proteins whose removal or destabilization lead to harmful phenotypic outcomes.

## 2 RESULTS

### 2.1 Constructing human SIs and mapping mutations

**FIGURE 2:**
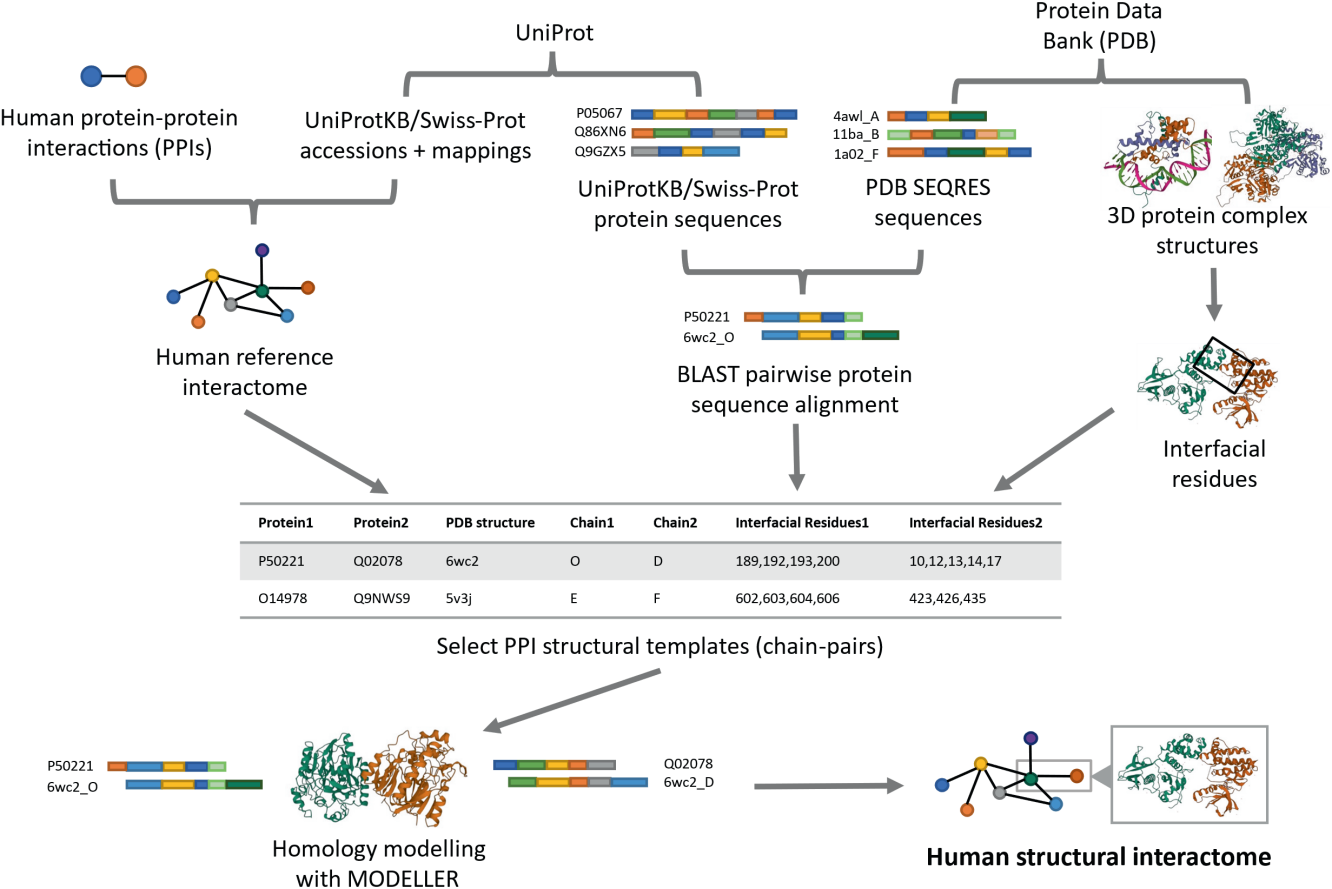
Computational pipeline for building a human structural interactome (SI) from human binary PPIs. Two SIs were constructed, HI‐union‐SI and IntAct‐SI, from the HI‐union (Luck et al., 2020) and IntAct (del Toro et al., 2022) databases, respectively. 3D structural models for PPIs were constructed through MODELLER (Webb & Sali, 2016), using sequences from the UniProtKB/Swiss‐Prot (The UniProt Consortium, 2023) database, SEQRES sequences and 3D structures from the Protein Data Bank (PDB) (Berman et al., 2000), and the BLAST (Camacho et al., 2009) protein alignment tool (BLASTP).

We constructed two human SIs, HI‐union‐SI and IntAct‐SI, consisting of 3D homology models for verified and high‐quality human binary PPIs from the HI‐union (Luck et al., 2020) and IntAct (del Toro et al., 2022) databases, respectively. Sequences of interacting proteins were retrieved from the UniProtKB/Swiss‐Prot (The UniProt Consortium, 2023) database. Sequences of interacting chains in 3D experimentally determined protein structures were obtained from the Protein Data Bank (PDB) (Berman et al., 2000). Binary interactions were verified using a sequence similarity approach to confirm that interacting protein pairs are homologous to chains in the same PDB protein complex as well as a distance‐based approach to confirm existing interfacial residues between protein chains. 3D homology models of PPIs were constructed by inputting BLASTP (Camacho et al., 2009) alignments of Swiss‐Prot proteins against homologous PDB chains into MODELLER (Webb & Sali, 2016). MODELLER was chosen for its versatility in constructing homology models from predefined structural templates (Akdel et al., 2022; Junk & Kiel, 2022; Larivière et al., 2012; Mosca et al., 2013; Xia et al., 2021; Yajima et al., 2024). Figure 2 depicts the complete pipeline for building our SIs. Our HI‐union‐SI consists of 2196 structural PPIs among 1705 Swiss‐Prot proteins, while our IntAct‐SI consists of 5499 structural PPIs among 4006 Swiss‐Prot proteins.

Although nonsynonymous mutations comprise nonstop mutations, we excluded nonstop mutations from our analysis because of (1) data scarcity and (2) given the nature of nonstop mutations (stop codon replaced by a non‐stop codon corresponding to an amino acid), they are unable to be mapped onto our SIs. Non‐pathogenic missense and nonsense mutations were curated from dbSNP (Sherry et al., 2001). Pathogenic Mendelian disease‐causing missense and nonsense mutations were retrieved from ClinVar (Landrum et al., 2016). Missense and nonsense mutations were mapped onto the Swiss‐Prot proteins in the HI‐union‐SI and IntAct‐SI using mRNA to protein mappings from RefSeq (O'Leary et al., 2016). For each SI, we retained mutations that are covered by at least one PPI structural model. The HI‐union‐SI consists of 8263 dbSNP and 445 ClinVar missense mutations and 165 dbSNP and 277 ClinVar nonsense mutations. The IntAct‐SI comprises 23320 dbSNP and 1363 ClinVar missense mutations and 421 dbSNP and 1434 ClinVar nonsense mutations.

### 2.2 Mendelian disease‐causing mutations are more likely to be quasi‐null or nonsense than non‐pathogenic mutations

**FIGURE 3:**
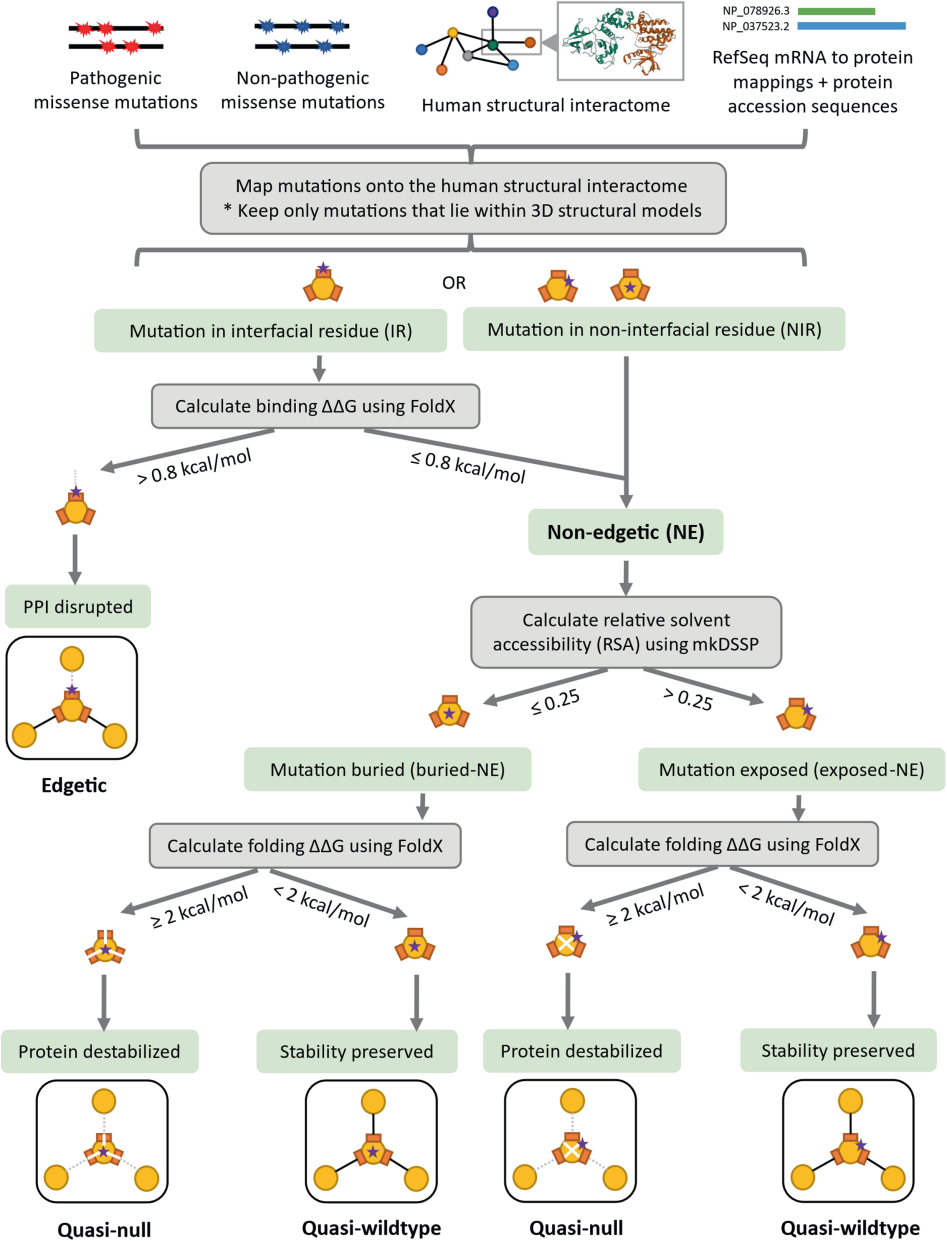
Computational pipeline for mapping missense mutations onto a structural interactome, determining their locations within the 3D protein structures, and predicting their edgotypes (quasi‐wildtype, edgetic, or quasi‐null). Mutations were mapped onto the HI‐union‐SI and IntAct‐SI using protein accession mappings and sequences from RefSeq (O'Leary et al., 2016). Edgotypes were predicted based on mutation locations, FoldX version 5 (Delgado et al., 2019, 2025; Schymkowitz et al., 2005) binding and folding ΔΔG calculations, and relative solvent accessibility (RSA) values (Kabsch & Sander, 1983; Sydykova et al., 2018). The binding ΔΔG threshold was derived from the FoldX estimated error (Guerois et al., 2002). Thresholds for folding ΔΔG and RSA were established by previous studies (Ghadie & Xia, 2019, 2021; Gong et al., 2017; Xu et al., 2012).

To elucidate disparities in the functional consequences of non‐pathogenic and Mendelian disease‐causing mutations, we compared the fractions of quasi‐null and nonsense mutations among the dbSNP and ClinVar datasets. We created datasets of nonsynonymous mutations by merging nonsense and missense mutations that mapped onto proteins in our SIs. In the HI‐union‐SI, 2.0% of dbSNP and 38.4% of ClinVar nonsynonymous mutations are nonsense (Figure 5b). Similarly, in the IntAct‐SI, 1.8% of dbSNP and 51.3% of ClinVar nonsynonymous mutations are nonsense. These observations indicate that Mendelian disease‐causing mutations are more likely to be nonsense than non‐pathogenic mutations. We predicted the edgotype (quasi‐wildtype, edgetic, or quasi‐null) of each missense mutation based on its location within the protein structure, its FoldX version 5 (Delgado et al., 2019; Delgado et al., 2025; Schymkowitz et al., 2005) binding and folding ΔΔG calculations, and its relative solvent accessibility (RSA). FoldX was employed for its widespread application in computational mutagenesis studies (Burke et al., 2023; Junk & Kiel, 2021; Lei et al., 2024; Sergeeva et al., 2020; Stephenson et al., 2024; Zhao et al., 2014). The complete pipeline is described in Figure 3. We defined a mutation to be quasi‐null if it is non‐edgetic (NE) (does not disrupt PPIs) and affects the stability of the protein (folding ΔΔG ≥2 kcal/mol). Additionally, many quasi‐null mutations are also buried in the core of the protein (RSA ≤0.25). We computed the mean BLOSUM62 scores (Henikoff & Henikoff, 1992) of the amino acid substitutions resulting in quasi‐wildtype, edgetic, and quasi‐null mutations (Figure S1a,b). In both SIs, we observed a progressive decrease in the mean BLOSUM62 score from quasi‐wildtype to edgetic to quasi‐null. This trend reflects the relative conservation of these missense mutations and provides validation for our edgotyping results. We further confirmed that our edgotyping results, obtained through structural systems biology approaches, are broadly consistent with those found using experimental assays (Sahni et al., 2015). Specifically, in both SIs, we found that ClinVar Mendelian disease‐causing missense mutations are more likely to be quasi‐null than dbSNP non‐pathogenic missense mutations. In the HI‐union‐SI, 6.5% of dbSNP and 19.3% of ClinVar missense mutations are quasi‐null. Similarly, in the IntAct‐SI, 7.5% of dbSNP and 25.2% of ClinVar missense mutations are quasi‐null (Figure 5a). Overall, our findings concur with the notion that disease mutations cause greater functional implications, in the form of node removals (loss‐of‐function or destabilization) in the protein interactome, than non‐pathogenic mutations (Mort et al., 2008; Redler et al., 2016).

### 2.3 Quasi‐null and nonsense mutations are evenly distributed across the first half and last half of proteins

**FIGURE 4:**
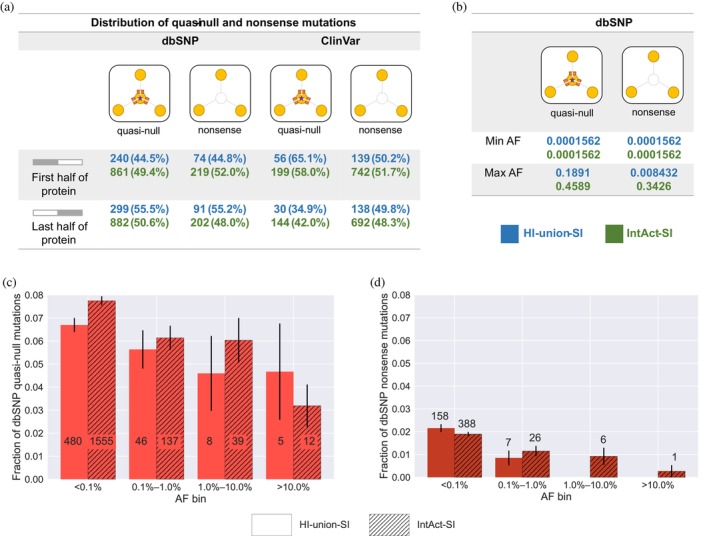
Comparing the positions and allele frequencies (AFs) of quasi‐null and nonsense mutations. AFs were obtained for dbSNP non‐pathogenic variants from the 1000 Genomes phase 3 project (Byrska‐Bishop et al., 2022). dbSNP mutations were categorized into four AF bins: <0.1%, 0.1%–1.0%, 1.0%–10.0%, and >10.0%. (a) Table presenting the numbers and percentages of dbSNP non‐pathogenic and ClinVar Mendelian disease‐causing quasi‐null and nonsense mutations distributed across either the first half or last half of the proteins in the HI‐union‐SI (blue) and IntAct‐SI (green). (b) Table displaying the minimum and maximum AFs of dbSNP quasi‐null and nonsense variants in proteins within the HI‐union‐SI (blue) and IntAct‐SI (green). (c) Fractions of dbSNP quasi‐null variants in proteins within the HI‐union‐SI (unhatched bars) and IntAct‐SI (hatched bars) across the four AF bins. The number on each bar indicates the number of quasi‐null variants in each bin. Error bars represent the standard error. (d) Fractions of dbSNP nonsense variants in proteins within the HI‐union‐SI (unhatched bars) and IntAct‐SI (hatched bars) across the four AF bins. The number on top of each bar indicates the number of nonsense variants in each bin. Error bars represent the standard error. The HI‐union‐SI contains nonsense mutations only in the two rarest AF bins (<0.1% and 0.1%–1.0%).

To gain insight into the correlation between the position of a quasi‐null or nonsense mutation in a protein and the resulting phenotypic outcomes, we compared the percentages of mutations in the first half and last half of proteins. Interestingly, in both SIs, approximately half of the ClinVar Mendelian disease‐causing quasi‐null and nonsense mutations are positioned in the first half of proteins (Figure 4a). The same trend is observed for the dbSNP non‐pathogenic quasi‐null and nonsense mutations. Our results indicate that quasi‐null and nonsense mutations are evenly distributed across the first half and last half of proteins, regardless of whether the mutations are non‐pathogenic or Mendelian disease‐causing.

### 2.4 Quasi‐null and nonsense variants are less prevalent at high allele frequencies

To better understand a variant's functional implications in the human population, we investigated the relationship between a population genetic measure (allele frequency) and a biophysical measure (fraction of node‐removal mutations in the interactome). We obtained allele frequencies (AFs) of dbSNP non‐pathogenic quasi‐null and nonsense mutations from the 1000 Genomes phase 3 project (Byrska‐Bishop et al., 2022). In both SIs, the minimum AFs of dbSNP quasi‐null and nonsense mutations are identical (0.0001562) (Figure 4b). For mutations in proteins within the HI‐union‐SI, the maximum AFs of dbSNP quasi‐null and nonsense mutations are 0.1891 and 0.008432, respectively. For mutations in proteins within the IntAct‐SI, the maximum AFs of dbSNP quasi‐null and nonsense mutations are 0.4589 and 0.3426, respectively. In both SIs, the maximum AF of dbSNP quasi‐null mutations is consistently larger than that of dbSNP nonsense mutations, suggesting that quasi‐null mutations are under more relaxed constraints in the population and may be less deleterious than nonsense mutations.

Next, we binned the dbSNP non‐pathogenic mutations in proteins within our SIs into four AF bins: <0.1%, 0.1%–1.0%, 1.0%–10.0%, and >10.0%. For each AF bin, we calculated the fractions of quasi‐null variants (# of quasi‐null mutations divided by total # of missense mutations) and nonsense variants (# of nonsense mutations divided by total # of nonsynonymous mutations). As mentioned previously, nonstop mutations were omitted from our dataset of nonsynonymous mutations. For both SIs, we found an overall decreasing trend in the fractions of quasi‐null and nonsense variants across the rarest AF bin (<0.1%) to the most common AF bin (>10.0%) (Figure 4c,d). This trend indicates that the relationship between AFs and the fractions of quasi‐null/nonsense variants follows a negative log‐linear trend. Overall, our findings indicate that, while the evolutionary pressures on quasi‐null and nonsense variants differ substantially in strength, they are qualitatively similar in nature (in terms of how these variants are distributed in the population).

### 2.5 There is a gap in deleteriousness between quasi‐null and nonsense mutations

**FIGURE 5:**
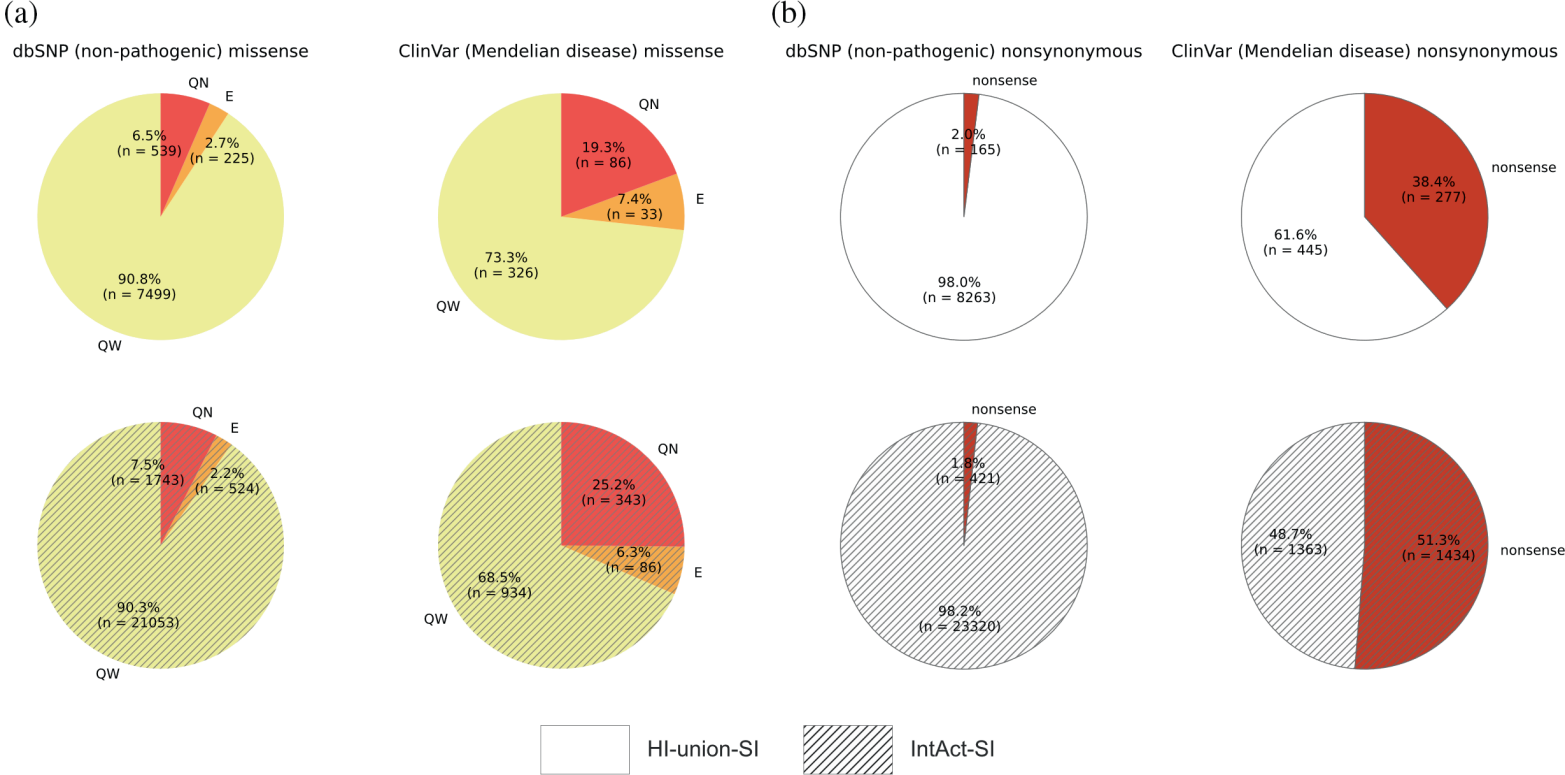
Evaluating quasi‐null and nonsense mutations through calculations of the fold difference between ClinVar Mendelian disease‐causing and dbSNP non‐pathogenic fractions. (a) Piecharts displaying the numbers and percentages of quasi‐wildtype (QW, yellow), edgetic (E, orange), and quasi‐null (QN, bright red) mutations among the dbSNP non‐pathogenic (left column) and ClinVar Mendelian disease‐causing (right column) missense mutations affecting proteins in the HI‐union‐SI (top row, unhatched) and IntAct‐SI (bottom row, hatched). (b) Piecharts displaying the numbers and percentages of nonsense mutations (dark red) among the dbSNP non‐pathogenic (left column) and ClinVar Mendelian disease‐causing (right column) nonsynonymous mutations affecting proteins in the HI‐union‐SI (top row, unhatched) and IntAct‐SI (bottom row, hatched). Nonsynonymous mutations included missense and nonsense mutations; nonstop mutations were excluded due to data scarcity and the inability to map them onto proteins in the SIs.

For a universal evaluation of the functional consequences of different types of node‐removal mutations (either quasi‐null or nonsense), we calculated their fold differences, defined as the ratio between the fractions of node‐removal mutations in datasets of ClinVar Mendelian disease‐causing and dbSNP non‐pathogenic mutations. Such calculations measure the degree of deleteriousness of Mendelian disease‐causing mutations compared to non‐pathogenic mutations and provide an assessment of the severities of quasi‐null and nonsense mutations. The fold differences between the fractions of ClinVar and dbSNP quasi‐null mutations in the HI‐union‐SI and IntAct‐SI are 19.3%/6.5% = 2.97 and 25.2%/7.5% = 3.36, respectively (Figure 5a). We repeated the same calculations for the fractions of ClinVar and dbSNP nonsense mutations in the HI‐union‐SI and IntAct‐SI, resulting in fold differences of 38.4%/2.0% = 19.2 and 51.3%/1.8% = 28.5, respectively (Figure 5b). Overall, quasi‐null mutations exhibit at least a 3‐fold difference, while nonsense mutations display at least a 20‐fold difference, suggesting that there is a disparity in deleteriousness between quasi‐null and nonsense mutations.

### 2.6 Quasi‐null mutations that cause greater folding instability (larger folding ΔΔG) are more functionally comparable to nonsense mutations

**FIGURE 6:**
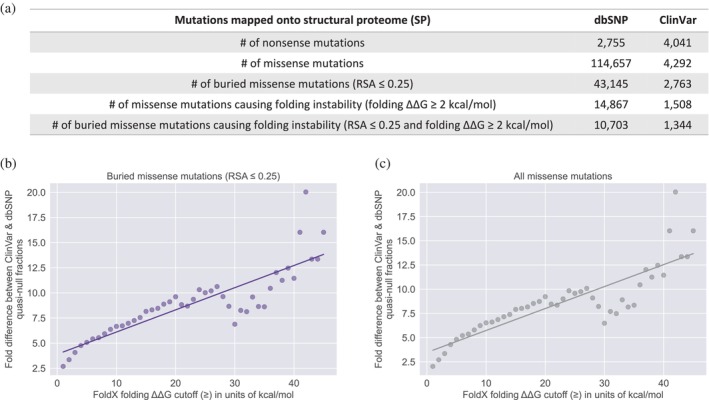
Assessing the range in fold differences of quasi‐null mutations mapped onto a structural proteome (SP) constructed from the UniProtKB/Swiss‐Prot human proteome. (a) Table depicting the numbers of nonsense mutations, missense mutations, buried missense mutations (RSA ≤0.25), missense mutations causing protein folding instability (folding ΔΔG ≥2 kcal/mol), and buried missense mutations causing folding instability (RSA ≤0.25 and folding ΔΔG ≥2 kcal/mol) within our supersets of dbSNP and ClinVar mutations. These supersets of nonsense and missense mutations were mapped onto our SP, instead of our two SIs. Plots portraying the positive linear correlation between fold differences (between ClinVar and dbSNP quasi‐null fractions) and folding ΔΔG cutoffs ranging from 1 kcal/mol (mildly unstable folding) to 45 kcal/mol (extremely unstable folding), for two possible definitions of quasi‐null mutations: (b) buried missense mutations (RSA ≤0.25) that meet a specific folding ΔΔG threshold and (c) all missense mutations that satisfy a certain folding ΔΔG cutoff, without considering RSA values.

To investigate the functional comparability of quasi‐null and nonsense mutations, we evaluated the impact of folding ΔΔG (the change in stability of a protein upon mutation) on the fold difference (between Mendelian disease‐causing and non‐pathogenic datasets) of quasi‐null mutations. We first constructed a structural proteome (SP) consisting of 3D homology models for proteins in the UniProtKB/Swiss‐Prot database (The UniProt Consortium, 2023). As the folding stability of a protein can be considered independently of its PPIs, our SP served as a resource for expanding our datasets of quasi‐null mutations. Unlike our SIs, our SP includes a broader range of proteins, as it is not restricted to those with confirmed interactions. As such, we created supersets of missense mutations by mapping mutations onto our SP. Of these missense mutations, ones that affect protein stability were further differentiated into supersets of quasi‐null mutations. Our original definition of a quasi‐null mutation is a NE (does not disrupt PPIs) mutation that (1) induces folding instability (folding ΔΔG ≥2 kcal/mol). Additionally, many quasi‐null mutations are also (2) buried in the core of the protein (RSA ≤0.25). Figure 6a depicts the number of all missense mutations as well as those meeting either one or both criteria for characterizing a quasi‐null mutation. We investigated whether changing the folding ΔΔG threshold for folding instability affects the fold difference between ClinVar Mendelian disease‐causing and dbSNP non‐pathogenic supersets of quasi‐null mutations. We found that the fold difference increases linearly with increasing folding ΔΔG threshold (1–45 kcal/mol), with a best fit line of *y* = 0.221*x* + 3.897 (Figure 6b). As nonsense mutations generally result in the absence of protein products, it is not possible to compute the thermodynamic free energy changes induced by these mutations. However, we estimate that on average, the deleteriousness of a nonsense mutation is similar to that of a severe quasi‐null mutation (with folding ΔΔG >40 kcal/mol). In summary, our results show that the larger the impact a quasi‐null mutation has on the folding stability of the protein, the more deleterious it is and the more comparable it is to a nonsense mutation. Such findings demonstrate that the functional deleteriousness of quasi‐null mutations resides on a continuous spectrum, with nonsense mutations positioned at the extreme (highly deleterious) end.

### 2.7 RSA is auxiliary in the definition of a quasi‐null mutation

The RSA of a residue, ranging from 1.0 (complete exposure) to 0.0 (complete burial), is a structural property that reflects the residue's microenvironment and degree of burial within the 3D protein structure. However, the relative importance of RSA and folding stability in characterizing quasi‐null mutations remains uncertain. As such, we assessed whether a quasi‐null mutation can be determined solely by its effect on the stability of the protein (folding ΔΔG), without considering its RSA. For missense mutations defined to be quasi‐null solely based on their folding ΔΔG values, we found that the fold difference (between the ClinVar Mendelian disease‐causing and dbSNP non‐pathogenic datasets) of quasi‐null mutations increases linearly with increasing folding ΔΔG threshold (1–45 kcal/mol), with a best fit line of *y* = 0.227*x* + 3.473 (Figure 6c). This best fit line is nearly identical to the one depicted in Figure 6b, suggesting that RSA is not the main defining feature of a quasi‐null mutation.

We further evaluated the relevance of RSA in characterizing a quasi‐null mutation by altering the RSA cutoff in two possible definitions of quasi‐null mutations: (1) missense mutations that induce folding instability (folding ΔΔG ≥2 kcal/mol) and satisfy a particular RSA cutoff (1.0 to 0.0) (Figure S2a) and (2) missense mutations that meet a certain RSA threshold (1.0 to 0.0), without considering folding ΔΔG values (Figure S2b). For definition (1), the maximum fold difference is approximately 4.5 when the quasi‐null mutations are completely buried. Whereas for definition (2), the maximum fold difference at complete burial is approximately 2.75, indicating that folding ΔΔG is more important than RSA in defining quasi‐null mutations. Moreover, for both definitions, the maximum fold difference does not reach 5 even for completely buried mutations, further suggesting that RSA values play a secondary role in designating quasi‐null mutations. Although RSA is important for evaluating the functional implications of a mutation, it is folding ΔΔG, rather than RSA, that is the key indicator of the mutation's direct effect on the protein.

### 2.8 Fold differences are not confounded by the coverage of the homology models

**FIGURE 7:**
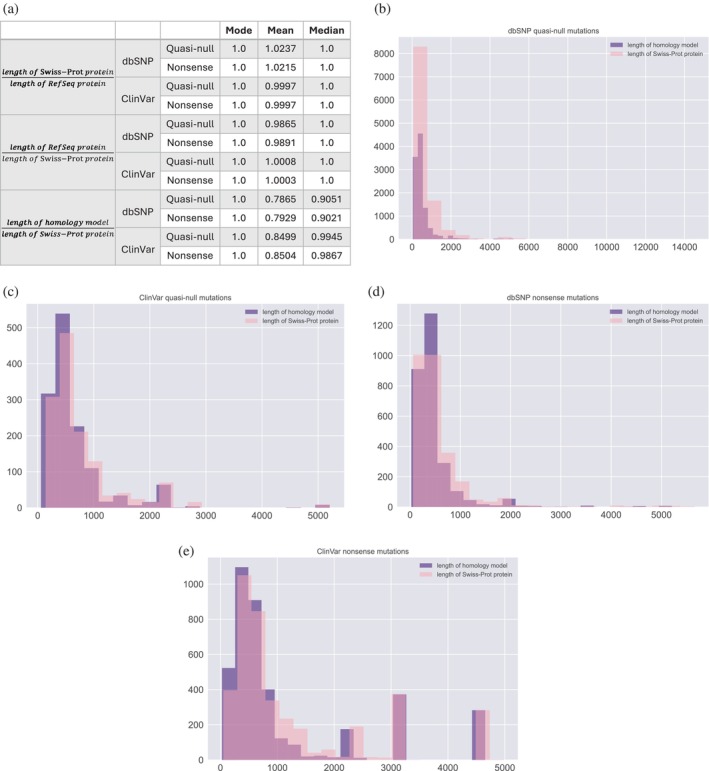
Investigating potential confounders that may influence fold difference and folding ΔΔG calculations using our supersets of dbSNP and ClinVar quasi‐null and nonsense mutations that were mapped onto our structural proteome. Here we defined a missense mutation to be quasi‐null if it has RSA ≤0.25 (buried in the core of the protein and thus more likely to be non‐edgetic) and folding ΔΔG ≥2 kcal/mol. (a) Table portraying the mean, median, and mode for three potential confounding factors: (1) length of Swiss‐Prot protein over length of RefSeq protein, (2) length of RefSeq protein over length of Swiss‐Prot protein, and (3) length of homology model over length of Swiss‐Prot protein. The mean, median, and mode values for (1) and (2) are all either 1.0 or nearly 1.0, indicating that discrepancies in the lengths of RefSeq and Swiss‐Prot proteins may not be a confounding factor. Quasi‐null and nonsense mutations were categorized into five bins for (3): (0.0, 0.2], (0.2, 0.4], (0.4, 0.6], (0.6, 0.8], (0.8, 1.0]. Plots illustrating the distributions of the lengths of the homology models and Swiss‐Prot proteins containing (b) dbSNP quasi‐null, (c) ClinVar quasi‐null, (d) dbSNP nonsense, and (e) ClinVar nonsense mutations.

We investigated possible confounders that may influence our fold difference calculations for measuring the functional deleteriousness of quasi‐null and nonsense mutations. Although the proteins in our HI‐union‐SI, IntAct‐SI, and SP were obtained from the UniProtKB/Swiss‐Prot (The UniProt Consortium, 2023) human proteome, the positions of dbSNP and ClinVar mutations were provided in coordinates along RefSeq transcripts and proteins. When mapping mutations onto our SIs and SP, we determined the positions of the mutations along the Swiss‐Prot proteins using the flanking sequences of the mutations in the RefSeq proteins. However, existing discrepancies in the lengths and positions of mutations along RefSeq and Swiss‐Prot protein sequences could potentially confound our fold difference results. As such, for each quasi‐null and nonsense mutation in our SP (Figure 6a), we calculated the ratio between the lengths of the RefSeq and Swiss‐Prot proteins affected by the mutation. As we are unable to determine whether a missense mutation is NE (does not disrupt PPIs) in our SP, we defined a missense mutation in our SP to be quasi‐null if it has RSA ≤0.25 (buried in the core of the protein and thus more likely to be NE) and folding ΔΔG ≥2 kcal/mol. We found that regardless of whether the length of the RefSeq protein or length of the Swiss‐Prot protein was used as the denominator, the modes and medians of the ratios for dbSNP and ClinVar quasi‐null and nonsense mutations were consistently 1.0 (Figure 7a). Furthermore, the averages of these ratios were also very close to 1.0, suggesting that the discrepancies in lengths between RefSeq and Swiss‐Prot proteins are not significant enough to confound our fold difference analyses.

**FIGURE 8:**
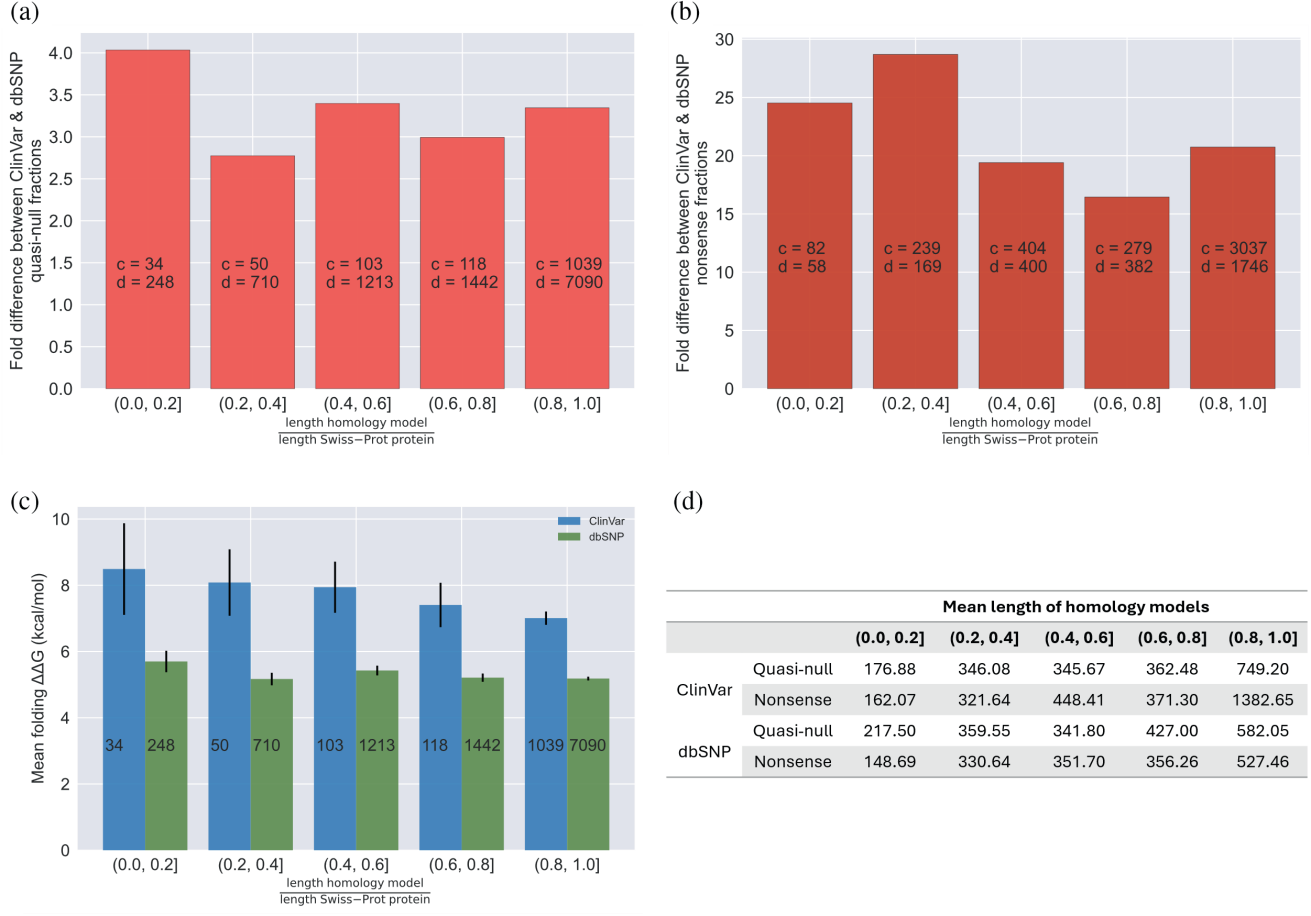
Exploring the potential for the fold differences of quasi‐null and nonsense mutations (mapped onto our structural proteome) to be confounded by the extent of the homology models' coverages of the Swiss‐Prot proteins the mutations occur in. For each quasi‐null and nonsense mutation, we calculated the ratio of the length of the homology model to the length of the Swiss‐Prot protein containing the mutation and categorized the mutation into five bins based on this ratio: (0.0, 0.2], (0.2, 0.4], (0.4, 0.6], (0.6, 0.8], (0.8, 1.0]. (a) Plot illustrating the fold difference between the ClinVar and dbSNP quasi‐null fractions across the five bins. The fold difference remains around 3 across each bin. In each bar, “*c*” and “*d*” represent the number of ClinVar and dbSNP quasi‐null mutations, respectively. (b) Plot depicting the fold difference between the ClinVar and dbSNP nonsense fractions across the five bins. The fold difference remains around 20 across each bin. In each bar, “*c*” and “*d*” represent the number of ClinVar and dbSNP nonsense mutations, respectively. (c) Plot illustrating the overall decrease in the mean folding ΔΔG of ClinVar (blue) and dbSNP (green) quasi‐null mutations across the five bins. The number in each bar indicates the number of quasi‐null mutations. (d) Table displaying the mean length of the homology models containing ClinVar and dbSNP quasi‐null and nonsense mutations across the five bins. The mean length generally increases with an increase in coverage of the homology models.

Lastly, we evaluated whether our fold difference and folding ΔΔG calculations for the dbSNP and ClinVar quasi‐null and nonsense mutations mapped onto our SP are confounded by the coverage of the 3D homology models constructed for the Swiss‐Prot proteins that contain these mutations. We plotted distributions of the lengths of the homology models and Swiss‐Prot proteins containing dbSNP quasi‐null (Figure 7b), ClinVar quasi‐null (Figure 7c), dbSNP nonsense (Figure 7d), and ClinVar nonsense (Figure 7e) mutations. For each of the quasi‐null and nonsense mutations in our SP, we calculated the ratio of the length of the homology model to the length of the Swiss‐Prot protein that the model represents. The averages and medians of these ratios for the dbSNP and ClinVar quasi‐null and nonsense mutations substantially deviated from 1.0 (Figure 7a), indicating that the coverage of the homology models is a possible confounding factor. Hence, we binned each quasi‐null and nonsense mutation into five categories based on the ratio (*r*) between the lengths of the homology model and Swiss‐Prot protein containing the mutation: 0.0 < *r* ≤ 0.2, 0.2 < *r* ≤ 0.4, 0.4 < *r* ≤ 0.6, 0.6 < *r* ≤ 0.8, 0.8 < *r* ≤ 1.0. The average length of the homology models in each of the five bins is a couple hundred residues (Figure 8d). Furthermore, even in the bin with the lowest coverage (coverage of the structural models is ≤0.2), the average length of the models is nonetheless greater than 100 residues, validating the effectiveness of our structural models. We observed that the fold differences between the ClinVar and dbSNP quasi‐null and nonsense fractions in each of the five bins persist around 3 and 20, respectively (Figure 8a,b), concurring with our estimates of the fold differences of quasi‐null (with folding ΔΔG ≥2 kcal/mol) and nonsense mutations. Our results indicate that fold differences are not confounded by the extent of the homology models' coverages of the corresponding Swiss‐Prot proteins. Interestingly, for dbSNP and ClinVar quasi‐null mutations, we observed an overall decreasing trend in the mean folding ΔΔG as well as a general increase in the mean length of the homology models across the bin with the least coverage (0.0 < *r* ≤ 0.2) to the bin with the most coverage (0.8 < *r* ≤ 1.0) (Figure 8c,d). Altogether, these two trends suggest that a quasi‐null mutation may induce larger folding instability (greater folding ΔΔG) when the homology model representing the protein is shorter in length.

### 2.9 Functional roles of dbSNP non‐pathogenic node‐removal mutations

To investigate the functional roles of node‐removal mutations in our dbSNP non‐pathogenic dataset, we compiled lists of the proteins in our SP (superset) that contain dbSNP quasi‐null and nonsense mutations for Reactome pathway enrichment analysis (Milacic et al., 2024). Within our lists, 3798 (out of 3817) Swiss‐Prot proteins containing dbSNP quasi‐null mutations and 1824 (out of 1836) Swiss‐Prot proteins containing dbSNP nonsense mutations were present in the Reactome pathway database. A substantial number (1361) of Swiss‐Prot proteins contain both dbSNP quasi‐null and nonsense mutations, highlighting a significant overlap between proteins impacted by destabilization and loss of function.

**FIGURE 9:**
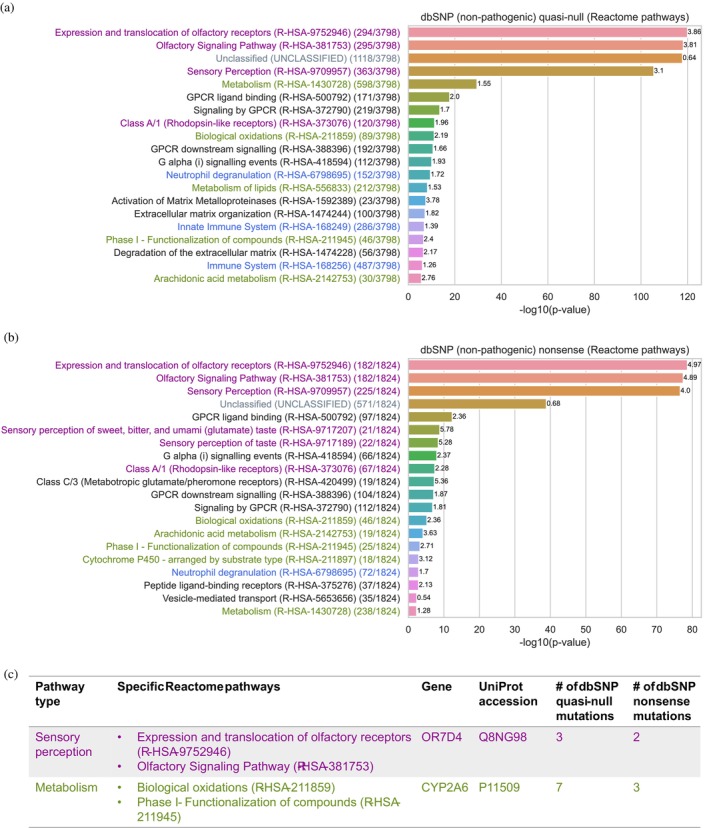
Investigating the functional roles of dbSNP non‐pathogenic node‐removal (quasi‐null or nonsense) mutations in the structural proteome. A total of 3798 (out of 3817) Swiss‐Prot proteins containing dbSNP quasi‐null mutations and 1824 (out of 1836) Swiss‐Prot proteins containing dbSNP nonsense proteins were present in the Reactome pathway database (Milacic et al., 2024). A total of 1361 Swiss‐Prot proteins were found to contain both dbSNP quasi‐null and nonsense mutations. Pathways are colored based on the following broad categories: sensory perception (purple), metabolism (green), immune system (blue), GPCR (G protein‐coupled receptor) signaling (black), and unclassified (gray). The fold enrichment of each pathway is displayed on top of the corresponding bar. The number of proteins found in each pathway is listed in parentheses next to the pathway name. Figures showing the top 20 most significant Reactome pathways of proteins containing dbSNP (a) quasi‐null and (b) nonsense mutations. (c) Table depicting a case study of a specific gene within each of the sensory perception (purple) and metabolism (green) categories.

Reactome pathway enrichment analysis of these proteins revealed that dbSNP node‐removal mutations (both quasi‐null and nonsense) are significantly enriched in proteins involved in sensory perception (purple), metabolism (green), immune system (blue), GPCR (G protein‐coupled receptor) signaling (black), and unclassified (gray) pathways (Figure 9a,b). GPCRs are broadly implicated in sensory perception, metabolism, and immune responses. The high degree of overlap between the top 20 pathways of proteins containing dbSNP quasi‐null mutations and the top 20 pathways of proteins containing dbSNP nonsense mutations emphasizes the functional similarity and comparability of protein destabilization and loss‐of‐function effects. Here, we discuss the overall effects of node‐removal mutations on the genes involved in the overlapping pathways within the following categories: sensory perception, metabolism, immune system, and unclassified. We further illustrate a case study of a specific gene within each of the sensory perception and metabolism categories.

Olfactory (smell) receptor genes are highly polymorphic, contributing to genetic diversity in individual perceptions of odor intensity and pleasantness (Trimmer et al., 2019). One such example is OR7D4 (UniProt accession: Q8NG98), an olfactory receptor gene found in the following sensory perception pathways: “Expression and translocation of olfactory receptors (R‐HSA‐9752946)” and “Olfactory Signaling Pathway (R‐HSA‐381753)”. OR7D4 contains three dbSNP quasi‐null and two dbSNP nonsense mutations in our SP (Figure 9c, shown in purple). Genetic variations in OR7D4 influence individual perceptions of androstenone, a steroid found in human secretions, which can be perceived as unpleasant (sweaty), pleasant (floral or sweet), or odorless (Araneda & Firestein, 2004; Wysocki & Beauchamp, 1984). Individuals with two functional copies of OR7D4 perceive androstenone as more unpleasant and more intense smelling than those carrying one or two non‐functional copies of OR7D4 (Hoover et al., 2015; Keller et al., 2007; Lunde et al., 2012). This example illustrates how node‐removal mutations in sensory perception pathways contribute to genetic diversity in sensory perception across the human population.

Genetic variants have been shown to contribute to metabolic diversity in human populations (Draisma et al., 2015; Illig et al., 2010; Shin et al., 2014; Zhou & Lauschke, 2022). A prominent example is CYP2A6 (UniProt accession: P11509), which contains seven dbSNP quasi‐null and three dbSNP nonsense mutations, and is involved in the following metabolic pathways: “Biological oxidations (R‐HSA‐211859)” and “Phase I ‐ Functionalization of compounds (R‐HSA‐211945)” (Figure 9c, shown in green). The CYP2A6 gene is highly polymorphic, resulting in individual differences in CYP2A6 enzymatic activity (Tanner & Tyndale, 2017; Zhou & Lauschke, 2022). As the CYP2A6 enzyme, a member of the cytochrome P450 superfamily of enzymes, is primarily responsible for the oxidation of nicotine and plays a crucial role in nicotine metabolism, mutations in the gene influence an individual's nicotine metabolism rate (Messina et al., 1997). Loss‐of‐function mutations that reduce or abolish CYP2A6 enzymatic activity result in slower nicotine metabolism, leading to higher blood nicotine levels that persist for a longer duration (Akrodou, 2015; Benowitz et al., 2006). As a result, individuals with these mutations have reduced nicotine cravings, a higher likelihood of quitting smoking or maintaining lower smoking rates, as well as a lower risk of developing lung cancer, compared to those with normal CYP2A6 enzymatic activity (Butler et al., 2021; Fujieda et al., 2004; Johani et al., 2020; Jones et al., 2021; Thorgeirsson et al., 2010; Wassenaar et al., 2015; Yuan et al., 2017). This example suggests that node‐removal mutations in metabolic pathways may contribute to individual variations in metabolic activity, fostering genetic heterogeneity that may be advantageous in certain situations.

The human immune system displays immense genetic diversity among individuals, enhancing the body's ability to defend against a wide range of pathogens (Liston et al., 2021). We observed that dbSNP quasi‐null mutations are significantly enriched in three immune‐related pathways, while nonsense mutations are enriched in just one, suggesting that quasi‐null mutations are more prevalent in immune system pathways than nonsense mutations (Figure 9a,b, shown in blue). This finding suggests that genes involved in immune system pathways are vital, while mutations causing relatively mild protein destabilization may be tolerated, as they could increase genetic variation and potentially enhance immune function.

Although the majority of the proteins with dbSNP quasi‐null and nonsense mutations are present in the Reactome pathway database, about one‐third of them (1118 out of 3798 for those with quasi‐null mutations and 571 out of 1824 for those with nonsense mutations) are enriched in the “Unclassified (UNCLASSIFIED)” pathway (Figure 9a,b, shown in gray). This indicates that many proteins with dbSNP non‐pathogenic node‐removal mutations are not well‐studied or well‐annotated, as expected.

Many factors could contribute to the tolerance of node‐removal mutations in certain proteins within the human population. These proteins may have redundant or non‐essential functions, have their function restored by compensatory mechanisms, have no observable phenotypic effects, or exhibit phenotypic effects that accumulate over time and become deleterious only at an advanced age. Future studies exploring these proteins in greater detail would enhance our understanding of the factors underlying this tolerance.

Overall, our preliminary analyses of the functional roles of dbSNP node‐removal mutations suggest that these mutations promote genetic variation in the human population, providing valuable insights into the mechanisms underlying the non‐pathogenic phenotypic outcomes of node‐removal mutations.

## 3 DISCUSSION

Nonsense mutations have been estimated to be twice as likely to result in a phenotype harmful enough to require medical attention than the most severe missense mutations (Krawczak et al., 1998). Such variations in the phenotypic consequences of missense and nonsense mutations have been linked to specific perturbations in the human protein interactome, the network of all PPIs within human cells (Sahni et al., 2013; Zhong et al., 2009). Although CADD (Kircher et al., 2014) has been introduced for estimating the deleteriousness of human genetic variants, it is incapable of explicitly distinguishing the functional outcomes of missense and nonsense alleles, limiting our understanding of the functional comparability of these mutations and how they contribute to phenotypic diversity and disease susceptibility. Thus, a method that considers the functional impacts of interactome perturbations will provide a more precise measure of the deleteriousness of missense and nonsense mutations. Here we employed structural systems biology approaches to construct high‐quality 3D structural models of binary human PPIs. We mapped missense and nonsense mutations onto these structural models and effectively distinguished a specific class of missense mutations, namely quasi‐null mutations, which exhibit comparable functional outcomes (node removal) to nonsense mutations. We calculated the effects of quasi‐null mutations on protein stability (folding ΔΔG) and formulated a novel method (fold difference) to quantitatively measure the change in the fractions of quasi‐null and nonsense mutations between datasets of Mendelian disease‐causing and non‐pathogenic mutations. Our fold difference calculations allow for a systematic and universal comparison of the functional and phenotypic effects of quasi‐null and nonsense mutations. Our results suggest that the deleteriousness of quasi‐null mutations spans a continuous spectrum, with nonsense mutations positioned at the extreme (highly deleterious) end. Such findings concur with the discrepancy in phenotypic severity observed between missense and nonsense mutations.

Quasi‐null mutations may be less deleterious than nonsense mutations due to the varying degrees to which these node‐removal mutations lead to protein misfolding and aggregation. A quasi‐null mutation, which induces folding instability through a single amino acid change in the resulting protein, may result in partial or global unfolding and aggregation of the mutated protein. However, since a quasi‐null mutation still produces a complete protein product, it is less likely to cause protein misfolding and aggregation to the same extent as a nonsense mutation. This is particularly true when the transcript of a nonsense mutation escapes NMD and results in a truncated protein. This truncated protein often lacks essential structural domains, which can prevent proper folding and expose hydrophobic regions, increasing the likelihood of severe misfolding and aggregation (Gough et al., 2012). Protein misfolding and aggregation often lead to cellular dysfunction and underlie many neurodegenerative diseases, such as Alzheimer's and highly infectious prion diseases, which trigger further protein misfolding and accumulation (Louros et al., 2023). Thus, quasi‐null mutations may result in less deleterious consequences than nonsense mutations because they are less likely to result in severe misfolding and aggregation. Future work on the molecular basis of protein misfolding and aggregation is crucial for a deeper understanding of the differences in selective pressures and deleterious effects between quasi‐null and nonsense mutations.

In addition, quasi‐null mutations, which destabilize the protein, may be less deleterious than nonsense mutations due to compensatory interactions that help stabilize the mutated protein. The functional impact of quasi‐null mutations on a protein is influenced by the nature of the interactions the protein participates in. Proteins that exist in large, stable complexes are involved in many permanent interactions, which can help mitigate the destabilizing effects of quasi‐null mutations. In such cases, the stability of the mutated protein is often compensated for by its interactions with other proteins in the complex. In contrast, a protein that primarily participates in transient interactions is more likely to be significantly impacted by quasi‐null mutations, as it lacks the stabilizing support of permanent binding partners. Thus, quasi‐null mutations in multi‐protein complexes are likely to be less harmful than nonsense mutations, which typically do not result in functional protein products. Future research on the effects of transient and permanent interactions on the functional impact of quasi‐null mutations would enhance our understanding of how protein stability is maintained and how these factors contribute to the differences in selective pressures and deleterious effects between quasi‐null and nonsense mutations.

The mutations investigated in this study, mutations with non‐pathogenic assertions from dbSNP (Sherry et al., 2001) and mutations causing Mendelian diseases from ClinVar (Landrum et al., 2016), were compiled from patient samples (in vivo). In vitro studies are able to assess the functional outcomes of wildtype and mutant proteins; however, the phenotypes observed in vitro may not appear in vivo (Khajavi et al., 2006). Thus, our investigation of mutations associated with in vivo phenotypes provides a direct and representative study of the naturally occurring genotype‐to‐phenotype connections in the human population.

### 3.1 Assessing the robustness of the structural models

We compared our structural and mutation edgotype predictions from MODELLER with those from AlphaFold3 (Abramson et al., 2024). We first obtained dimers (PPIs) that exist in both our HI‐union‐SI and IntAct‐SI and selected those containing mutations across all three edgotypes (quasi‐wildtype, edgetic, and quasi‐null). Among the dimers with mutations spanning all three edgotypes, we randomly selected 10 dimers, consisting of 20 unique monomers, for AlphaFold3 dimer structure predictions. These 10 dimers contain 26 ClinVar mutations and 74 dbSNP mutations. We used the PyMOL (Schrödinger, LLC, 2025) super command to calculate the RMSDs between the 10 dimers (and their corresponding 20 monomers) modeled by MODELLER and their respective counterparts predicted by AlphaFold3 (Figure S3a). Overall, there is an excellent agreement between the structural models predicted by MODELLER and AlphaFold3 for these 10 dimers. Specifically, nine out of the 10 dimers had low RMSDs (≤4.5 Å) between the MODELLER and AlphaFold3 predictions, indicating that overall, the MODELLER and AlphaFold3 dimer structure predictions are similar to each other.

The only exception is the dimer (Q9NRX1, Q9ULX3) with high RMSD (10.3 Å) between MODELLER and AlphaFold3 predictions (Figure S3a). In this case, the dimer had low AlphaFold3 pTM (0.39) and ipTM (0.40) scores, suggesting that the AlphaFold3 prediction of the overall structure and dimer interface is of low confidence. Since the monomers in the same dimer modeled by MODELLER had 100% sequence identities (excluding gaps) to the PDB template 7wu0_x and 7wu0_y chains, the MODELLER‐based homology modeling prediction is likely to be close to the true structure. In support of this claim, the RMSD between the MODELLER dimer model and its template PDB chain‐pair (7wu0_x and 7wu0_y) is 2.9 Å, while the RMSD between the AlphaFold3 dimer model and the same PDB chain‐pair is 8.3 Å. Altogether, these findings suggest that for this particular dimer (Q9NRX1, Q9ULX3), the disagreement between the MODELLER and AlphaFold3 structural models is likely due to the low quality of the AlphaFold3 prediction.

To further compare the mutation effects between MODELLER and AlphaFold3 predicted structures, we used FoldX version 5 (Delgado et al., 2019, 2025) to calculate the effects of the mutations on the binding ΔΔG and folding ΔΔG of the AlphaFold3 structures. We predicted a mutation to disrupt an interaction if the binding ΔΔG >0.8 kcal/mol, and a mutation to disrupt the folding stability of a protein if the folding ΔΔG ≥2 kcal/mol. Such thermodynamic energy calculations and thresholds were used to predict the edgotypes of these mutations. We compared the edgotypes of the mutations in our MODELLER models with the edgotypes of the same mutations in the AlphaFold3 models. We termed a mutation “E → NE” (edgetic to non‐edgetic) if it disrupted the interaction between the monomers in our MODELLER dimer but not in the corresponding AlphaFold3 dimer. We labeled a mutation “QN → QW” (quasi‐null to quasi‐wildtype) if it disrupted the stability of the monomer in the MODELLER dimer but not in the corresponding AlphaFold3 dimer. We designated a mutation “QW → QN” (quasi‐wildtype to quasi‐null) if it did not disrupt the stability of the monomer in the MODELLER dimer but did in the corresponding AlphaFold3 dimer.

We observed that only a total of seven mutations out of the 100 mutations compared were either QN → QW or QW → QN (Figure S3b), indicating that there is only a small difference (7%) in the edgotype predictions of monomer folding stability between the MODELLER and AlphaFold3 models. The sole QN → QW mutation had folding ΔΔG of 1.2 kcal/mol (on the AlphaFold3 model), which does not deviate much from the ≥2 kcal/mol cutoff for a QN prediction. Moreover, the six QW → QN mutations had folding ΔΔG ranging between 2.1 and 3.2 kcal/mol (on the corresponding AlphaFold3 models), which also do not differ much from the <2 kcal/mol cutoff for a QW prediction. Thus, given this minor discrepancy in folding stability predictions, our monomer‐based edgotyping analyses from which our results and conclusions are drawn upon remain consistent regardless of whether MODELLER or AlphaFold3 is used for structural modeling.

In addition, we found that only a total of four mutations out of the 100 mutations compared were E → NE (Figure S3b), suggesting that there is only a small difference (4%) in the edgotype predictions of binding stability between the MODELLER and AlphaFold3 models. Hence, given this minor difference in binding stability predictions, our dimer‐based edgotyping analyses from which our results and conclusions are drawn upon remain consistent regardless of whether MODELLER or AlphaFold3 is used for structural modeling.

Overall, our preliminary findings suggest that MODELLER and AlphaFold3 predictions are structurally comparable and result in similar mutation edgotype predictions, aligning with expectations that AlphaFold3 performs similarly to MODELLER‐based homology modeling when applied to sequences with known close homologs in the PDB. Indeed, in the current study, we only include in our analysis either experimental crystal structures or MODELLER‐based homology models for sequences with known close homologs in the PDB. Further studies are necessary for a more comprehensive and definitive comparison between MODELLER and AlphaFold3.

### 3.2 Mutations in the tail ends of proteins

To gain insight into the structural and functional importance of residues in the tail ends of proteins, we investigated the distributions of dbSNP non‐pathogenic and ClinVar Mendelian disease‐causing quasi‐null and nonsense mutations across the first 5% and last 5% of proteins (Figure S4). In both SIs (HI‐union‐SI and IntAct‐SI), we observed that missense mutations in the first 5% and last 5% of proteins are generally less likely to be quasi‐null compared to those occurring throughout the entire sequences of proteins (Figure S4a). This observation aligns with the expectation that, on average, the first and last residues of a protein are less important in that they contribute less to the protein's stability, compared to residues in other regions. Hence, missense mutations in these ends are less likely to destabilize the protein and, therefore, less likely to be quasi‐null mutations. In addition, in both SIs, we observed that in the first 5% of proteins, there is a larger enrichment of ClinVar nonsense mutations than dbSNP nonsense mutations (Figure S4b). In contrast, in the last 5% of proteins, there is a larger enrichment of dbSNP nonsense mutations than ClinVar nonsense mutations (Figure S4b). These observations suggest that nonsense mutations in the first 5% of a protein are more likely to be deleterious, as most of the transcript is truncated, while those in the last 5% of a protein are more likely to be benign, as most of the transcript is still intact. However, our mutation data is limited and future studies using larger datasets would enable more comprehensive investigations of the structural and functional importance of residues in the first 5% and last 5% of proteins.

### 3.3 Impact of protein function on the deleteriousness of mutations

To better understand how protein function influences the deleteriousness of mutations, we investigated the following two protein functional categorizations: TF versus non‐TF, and housekeeping versus non‐housekeeping. We obtained 1469 Swiss‐Prot reviewed TF proteins annotated with “DNA‐binding transcription factor activity (GO:0003700)” from UniProtKB (The UniProt Consortium, 2023) and 9473 Swiss‐Prot reviewed housekeeping proteins from the Human Protein Atlas (Uhlén et al., 2015). Swiss‐Prot proteins not labeled as TFs were designated non‐TFs. Similarly, Swiss‐Prot proteins not labeled as housekeeping proteins were designated non‐housekeeping proteins. For each functional categorization, we divided the proteins in our SIs (HI‐union‐SI and IntAct‐SI) and SP into two groups, resulting in the following comparisons: (1) TF versus non‐TF and (2) housekeeping versus non‐housekeeping.

We observed that ClinVar Mendelian disease‐causing mutations are more likely to be quasi‐null or nonsense compared to dbSNP non‐pathogenic mutations, irrespective of whether they occur in TFs (Figure S5a,b), non‐TFs (Figure S5c,d), housekeeping (Figure S6a,b), or non‐housekeeping (Figure S6c,d) proteins. Our conclusion that Mendelian disease‐causing mutations cause greater functional implications (in the form of node removals in the protein interactome) than non‐pathogenic mutations remains consistent, regardless of the functional category of the mutated proteins.

Next, we calculated the fold differences between the fractions of ClinVar and dbSNP node‐removal mutations in proteins with different functional categories across our HI‐union‐SI and IntAct‐SI, and estimated the “Overall” fold differences derived from the two SIs (Figure S7). We observed a larger contrast in the fold differences of node‐removal mutations between housekeeping and non‐housekeeping proteins (Figure S7b) than between TF and non‐TF proteins (Figure S7a). For instance, the fold differences of node‐removal mutations (both quasi‐null and nonsense) in TF and non‐TF proteins are similar to each other, suggesting that the different types of specialized roles that TFs (regulatory roles) and non‐TFs (non‐regulatory roles) fulfill in the cell have comparable phenotypic importance. In contrast, the fold differences of nonsense mutations in housekeeping and non‐housekeeping proteins are 30 and 10, respectively (Figure S7b), emphasizing the functional importance of housekeeping proteins in maintaining fundamental cellular functions and cellular viability (Joshi et al., 2022; Tung et al., 2024). The three‐fold increase in fold differences of nonsense mutations observed from non‐housekeeping to housekeeping proteins further demonstrates the effectiveness of our fold difference measure in assessing the functional deleteriousness of mutations.

We further investigated the relationship between the phenotypic deleteriousness (fold difference) and folding instability (FoldX folding ΔΔG) of quasi‐null mutations across the two functional categorizations (Figures S8 and S9). We observed that in TFs, quasi‐null mutations tend to approach the deleteriousness of nonsense mutations (fold difference of 15) when the folding ΔΔG is ≥8 kcal/mol (Figure S8b,c), suggesting that TFs become non‐functional with relatively mild destabilization. TFs regulate gene expression by binding to DNA and other proteins through their DNA‐binding and activation domains. These domains adopt specific conformational states essential for effective binding and regulation; consequently, even small disruptions in stability can impair binding and result in a loss of regulatory function. Moreover, we found that in housekeeping proteins, quasi‐null mutations are significantly less deleterious than nonsense mutations (fold difference of 30), even at relatively high folding ΔΔG thresholds (Figure S9b,c). This suggests that many housekeeping proteins can tolerate substantial destabilization while remaining functional, likely due to their high expression levels and functional robustness. Thus, the activity of a housekeeping protein is unlikely to be completely abolished by a single missense mutation. In contrast, we observed that quasi‐null mutations in non‐TF (Figure S8d,e) and non‐housekeeping (Figure S9d,e) proteins follow trends similar to those seen across all proteins (Figure 6).

Overall, our conclusion that quasi‐null mutations causing greater folding instability (larger folding ΔΔG) are more functionally comparable to nonsense mutations remains consistent irrespective of the functional category of the affected proteins. Our preliminary analyses of protein functional categorization are limited by dataset size (Figures S5–S7, S8a, and S9a), particularly for mutations in TFs, and would greatly benefit from larger mutation datasets for more comprehensive and definitive analyses. Future studies investigating the relationship between protein functional categorization and the impact of node‐removal mutations would offer deeper insights into protein function and genotype‐to‐phenotype relationships.

### 3.4 Impact of protein–protein interaction types on the deleteriousness of mutations

In our study, we investigated solely direct physical interactions between proteins. These interactions, obtained from the HI‐union (Luck et al., 2020) and IntAct (del Toro et al., 2022) databases, have been validated at least twice experimentally or in literature. Such interactions can be differentiated based on their temporal or tissue‐specific nature, such as transient versus permanent or tissue‐specific versus non‐tissue‐specific. We classified the PPIs affected by our edgetic (edge loss) mutations to explore how disrupting different types of physical interactions influences the functional and phenotypic severity of these mutations.

We obtained transient and permanent PPIs from a recent study (Ghadie & Xia, 2022). These transient and permanent classifications were derived from 63 datasets of human gene time‐course expression levels (Barrett et al., 2013). We divided our edgetic mutations into the following three categories based on the temporal nature of the PPIs they disrupt: E1 (disrupts only transient PPIs), E2 (disrupts only permanent PPIs), and E3 (disrupts PPIs that are not clearly defined as either transient or permanent) (Figure S10a).

We obtained tissue‐specific and non‐tissue‐specific PPIs from a recent study (Ghadie & Xia, 2022). These tissue‐specific and non‐tissue‐specific classifications were derived from gene tissue expression data retrieved from the Illumina Body Map 2.0 project (Illumina, Inc., 2011), which consists of RNA‐seq data across 16 human tissue types. We divided our edgetic mutations into the following three categories based on the tissue‐specific nature of the PPIs they disrupt: E1 (disrupts only tissue‐specific PPIs), E2 (disrupts only non‐tissue‐specific PPIs), and E3 (disrupts PPIs that are not clearly defined as either tissue‐specific or non‐tissue‐specific) (Figure S10b).

For both PPI categorizations (transient versus permanent and tissue‐specific versus non‐tissue‐specific), we observed larger percentages of E1, E2, and E3 mutations in ClinVar Mendelian disease‐causing missense mutations than dbSNP non‐pathogenic missense mutations (Figure S10). These preliminary findings suggest that disease‐causing mutations are enriched in edgetic mutations compared to non‐pathogenic mutations, regardless of the temporal or tissue‐specific nature of the PPIs disrupted. Overall, our conclusion that Mendelian disease‐causing mutations cause greater functional implications (in the form of edge removals in the protein interactome) than non‐pathogenic mutations remains consistent. Future studies investigating the effects of disrupting transient versus permanent interactions on the functional and phenotypic deleteriousness of edgetic mutations are essential in advancing our understanding of the intricate mechanisms underlying genotype‐to‐phenotype relationships. Moreover, future studies addressing the influence of environmental factors, cellular conditions, and tissue‐specific requirements on network remodeling are crucial and will provide a more comprehensive understanding of the functional effects of the dynamic nature of PPIs.

### 3.5 Limitations

Theoretically, interactome perturbations comprise gain of edges (interactions) and/or nodes (proteins), in addition to edge/node losses. If the transcript of a nonsense mutation escapes NMD, the resulting truncated protein may have different binding partners than the full‐length protein, leading to possible edge and node gains. Furthermore, the missense mutations that we predicted to be “quasi‐wildtype” may have new binding sites in addition to their original binding sites. This could also be the case with nonstop mutations, a type of nonsynonymous mutation that produces longer‐than‐normal protein products, which were excluded from our analysis due to data scarcity. However, in this study we investigated solely edge and node losses in the protein interactome. Future studies exploring possible gain of interactions would enable a more comprehensive and inclusive study.

In our study, we assume that a missense mutation residing outside of a PPI interface does not disrupt the corresponding interaction. A mutation can allosterically affect the binding affinity or folding stability of the protein. However, it is difficult to accurately model allosteric effects, and traditional computational mutagenesis tools are not designed for explicitly calculating the free energy changes caused by long‐distance mutations. Thus, our edgotyping predictions focus exclusively on the consequences of local mutations. Further studies on long‐distance mutations will enable a broader and more encompassing investigation of the functional and deleterious effects of missense and nonsense mutations.

### 3.6 Future directions

Advancements in AI and machine learning models, such as large language models and AlphaFold3 (Abramson et al., 2024), have contributed significantly to predictions of protein 3D structure and PPIs. Specifically, AlphaFold3 can be employed to predict structures of dimers (PPIs) that lack closely related homologs in the PDB. These AlphaFold3 structural predictions could help increase the number of structural PPIs in our SIs, allowing for mutation edgotype predictions on a wider range of proteins, and providing more accurate and more comprehensive measurements of the fold differences of quasi‐null and nonsense mutations. Thus, future studies combining our current mutagenesis analyses and findings with these additional predictions can offer valuable insights into how genetic variants affect protein function, enhancing the assessment of their phenotypic impacts and potential deleteriousness, especially when integrated with orthogonal information such as evolutionary conservation.

Only a portion of ClinVar Mendelian disease‐causing missense mutations can be explained by edge (edgetic) and node (quasi‐null) losses in the protein interactome. Those predicted to be “quasi‐wildtype” likely cause diseases through other mechanisms, such as epistasis, gain of interactions, or disruptions of different types of molecular interactions. Epistasis refers to a genetic interaction between two mutations, where the effect of one mutation is altered—either suppressed, masked, or enhanced—by the presence of another mutation (the “epistatic” mutation) (Cordell, 2002). It is possible that the phenotypic effects of quasi‐wildtype mutations are influenced by the presence of epistatic mutations. In addition, quasi‐wildtype mutations may contribute to disease phenotypes through gain‐of‐interactions in the protein interactome, or by gaining or perturbing other types of molecular interactions. In particular, perturbations of protein–DNA, protein–RNA, and protein–metabolite interactions have been shown to cause diseases (Carpten et al., 2007; Lee et al., 2006; Sahni et al., 2015). A prominent example is missense mutations in the TCF4 protein, which impair DNA binding and lead to Pitt‐Hopkins Syndrome (Sirp et al., 2021). Future studies exploring potential epistatic mutations as well as mutation effects on interactions between proteins and other molecules will allow for a deeper and more encompassing understanding of disease mechanisms and genotype‐to‐phenotype relationships.

## 4 CONCLUSIONS

In conclusion, our universal “fold difference” measure provides a simple approach for quantifying and comparing the severity of node‐removal mutations (quasi‐null and nonsense). This measure may also be applied to edge‐removal mutations (edgetic) and potentially to other types of coding mutations to quantify their deleterious effects. Our observation of the strong positive correlation between biophysical destabilization and phenotypic deleteriousness suggests that mutation‐induced protein destabilization is indicative of the phenotypic outcomes of missense mutations. Our structural and thermodynamic analyses of node‐removal mutations allow for the potential identification of key proteins whose removal or destabilization lead to disease phenotypes, enabling the development of targeted therapeutic approaches. Overall, our study enhances comprehension of the intricate mechanisms governing genotype‐to‐phenotype relationships in clinically relevant single‐gene diseases.

## 5 MATERIALS AND METHODS

### 5.1 Constructing human reference interactomes

We obtained binary human PPIs from the HI‐union (Luck et al., 2020) and IntAct (del Toro et al., 2022) reference maps. PPIs in the HI‐union map were identified by yeast two‐hybrid assays while those in the IntAct database were curated from literature. As the interaction pairs in HI‐union are listed as Ensembl (Martin et al., 2023) gene IDs, we employed the UniProt (The UniProt Consortium, 2023) Human ID mappings file to convert the Ensembl gene IDs to corresponding UniProt protein accessions. For each Ensembl gene ID that mapped to more than one UniProt accession, we selected the UniProt accession with the longest protein sequence. We retrieved exclusively the three major types of physical interactions in the IntAct database: direct interactions (among proteins in direct contact), physical associations (among proteins in the same physical complex), and associations (among proteins that may form one or more physical complexes). We further selected for physical interactions that were reported at least twice in literature. As IntAct provides corresponding UniProt accessions, no gene to protein mapping was necessary. For each reference map, we retained non‐self‐interactions among UniProt proteins listed in the UniProtKB/Swiss‐Prot database (The UniProt Consortium, 2023) of high‐quality, reviewed, annotated, and non‐redundant proteins. These proteins are referred to as “Swiss‐Prot” proteins. PPIs among Swiss‐Prot proteins in the HI‐union and IntAct reference maps were used to construct the HI‐union and IntAct reference interactomes, respectively.

### 5.2 Building human SIs

Given that the majority of known PPIs lack experimentally determined 3D structures, we employed homology modeling, with the assistance of the BLAST protein alignment tool (BLASTP) (Camacho et al., 2009) and the MODELLER software (Webb & Sali, 2016), to computationally model 3D structures of PPIs. We obtained 3D structures of protein complexes at atomic‐level resolutions and their corresponding SEQRES chain sequences from the Protein Data Bank (PDB) (Berman et al., 2000) (Figure 2). To identify homologous chain sequences in each reference interactome, we used BLASTP to align the sequences of the Swiss‐Prot proteins in the interactome against all the PDB chain sequences in the PDB SEQRES records. We retained high‐quality alignments with E‐value <10^−5^. For each pair of Swiss‐Prot protein and PDB chain that had more than one possible alignment, the alignment with the smallest E‐value was selected. For each binary Swiss‐Prot PPI in the interactome, we compared the proteins' aligned PDB chains and retained plausible interacting pairs of PDB chains coming from the same structure. For each PDB chain‐pair, we extracted atomic‐level coordinates of the amino acid residues from the mmCIF file of their PDB structure and calculated the Euclidean distance between each residue of one PDB chain and all residues of the other PDB chain. We listed a residue as an “interfacial residue” (interacting residue that mediates the PPI) if any atom in this residue was within 5 Å of any atoms in any residues of the other chain. The interfacial residues on a structural chain collectively made up the PPI binding interface on the protein. We annotated a PPI with a structural chain‐pair if (1) the chains were from the same PDB structure, (2) there was at least one interfacial residue mediating their interaction, and (3) the corresponding structural chain for each Swiss‐Prot protein in the PPI had ≥50% of its interfacial residues aligned to the Swiss‐Prot protein sequence. For each PPI with multiple structural chain‐pair annotations, we selected one chain‐pair based on the resolution of the PDB structure (≤3.5 Å), the BLASTP alignment bitscore (≥50 and larger is preferred), and finally the number of interfacial residues (more is preferred). These selected PPI structural chain‐pairs served as templates for building homology models, and we inputted their corresponding BLASTP alignments into MODELLER to construct 3D structural models for the PPIs. As the structural models built by MODELLER are based on Swiss‐Prot protein sequences, we were able to map mutations onto the models accurately, without accounting for mismatching residues between the Swiss‐Prot and PDB SEQRES sequences. The resulting 3D PPI structural models of the HI‐union and IntAct reference interactomes collectively made up our HI‐union‐SI and IntAct‐SI, respectively.

### 5.3 Processing Mendelian disease‐causing and non‐pathogenic mutations

We obtained missense and nonsense mutations from the ClinVar database of clinically significant variants (Landrum et al., 2016) and the dbSNP database of molecular polymorphisms (Sherry et al., 2001). From the ClinVar database, we selected germline missense and nonsense mutations in the GRCh38 genome assembly that were directly labeled as “pathogenic” (i.e., Mendelian disease‐causing) with supporting evidence and no conflicting phenotypic interpretations. From the dbSNP database, we selected non‐pathogenic variants labeled as missense or nonsense, validated, not withdrawn, and without the following assertions: “pathogenic,” “likely pathogenic,” and “drug‐response.” Additionally, we obtained AFs of the dbSNP non‐pathogenic mutations from the 1000 Genomes phase 3 project (Byrska‐Bishop et al., 2022).

### 5.4 Mapping mutations onto human SIs

We mapped ClinVar Mendelian disease‐causing and dbSNP non‐pathogenic missense and nonsense mutations onto the HI‐union‐SI and IntAct‐SI (Figure 3). We first retrieved mRNA to protein accession mappings and sequences of protein accessions from RefSeq (O'Leary et al., 2016). We discarded mutations without corresponding mRNA‐protein mappings or protein accession sequences. For each mutation, we acquired its flanking sequence, the adjacent 10 amino acids (or all amino acids, if there are less than 10) on either side of the mutation, from its corresponding RefSeq protein sequence. These mutation flanking sequences were used to map the mutations onto their corresponding Swiss‐Prot protein sequences in the HI‐union‐SI and IntAct‐SI. If a mutation's flanking sequence had an exact (100%) match to a section of a Swiss‐Prot protein sequence, we kept the mutation for further analysis and noted down its position in the Swiss‐Prot protein. Previous studies have discarded any mutation whose position in the Swiss‐Prot protein is different from the position reported by either ClinVar or dbSNP (Ghadie & Xia, 2019, 2021); however, we kept these mutations and assumed that any position discrepancies are due to minor differences in insertions and deletions between the RefSeq and UniProt sequence databases. For each of the SIs, if the position of a mutation in a Swiss‐Prot protein was not found in at least one of the 3D structural models of the PPIs in which the protein participates we assumed that the mutation position was not structurally resolved and discarded the mutation. Additionally, within each of the two SIs, one mutation was selected for each unique amino acid change at any given position in the Swiss‐Prot proteins.

### 5.5 Predicting the edgotypes of missense mutations

To predict the edgotypes of missense mutations, we determined the locations of the mutations within the protein structures, the change in binding and folding free energies induced by the mutations, and the RSA values (Figure 3) (Ghadie & Xia, 2021). We categorized the mutations based on their locations in the 3D structural models of the PPIs in our two SIs (HI‐union‐SI and IntAct‐SI). If the position of a missense mutation in a protein coincided with one of the interfacial residues of any one of the protein's PPIs, we deemed the mutation to be located in an “interfacial residue (IR).” On the other hand, if the position of the missense mutation did not coincide with any of the interfacial residues of any of the protein's PPIs, we labeled the mutation as in “non‐interfacial residue (NIR).”

We predicted mutation edgotypes (edgetic or non‐edgetic) by combining our results of mutation locations in protein structures with thermodynamic binding energy (binding ΔΔG) calculations (Ghadie & Xia, 2021). For each IR mutation, we calculated the impact that it has on the binding ΔΔG of the PPIs that the protein participates in, using the FoldX version 5 Pssm command with default parameters (Delgado et al., 2019, 2025; Schymkowitz et al., 2005). A positive binding ΔΔG indicates that the PPI is destabilized (disrupted), while a negative binding ΔΔG indicates that the PPI is stabilized (preserved). Since the FoldX estimated ΔΔG error is 0.8 kcal/mol (Delgado et al., 2025; Guerois et al., 2002), we predicted an IR mutation to disrupt a PPI if it causes the binding ΔΔG of the PPI to be >0.8 kcal/mol. If an IR mutation is predicted to disrupt one or more PPIs, we labeled the mutation as “edgetic” (Sahni et al., 2013; Zhong et al., 2009). On the other hand, if an IR mutation causes the binding ΔΔG of all the PPIs the protein participates in to be ≤0.8 kcal/mol, we predicted that the mutation does not disrupt PPIs and deemed it “non‐edgetic (NE)” (Sahni et al., 2013; Zhong et al., 2009). We also predicted NIR mutations to be NE.

We further categorized NE mutations as “quasi‐wildtype” or “quasi‐null” (Sahni et al., 2015) by combining the RSA values of the mutations with thermodynamic folding energy (folding ΔΔG) calculations. To determine whether an NE mutation is exposed on the surface of the protein or buried in the core of the protein, we calculated the RSA of the residue using a procedure introduced by Sydykova et al. (2018) which employs the mkDSSP software (Kabsch & Sander, 1983). If the RSA of a residue is >0.25, we predicted the NE mutation to be exposed (exposed‐NE); otherwise, we predicted it to be buried (buried‐NE) (Ghadie & Xia, 2021; Gong et al., 2017; Xu et al., 2012). We further calculated the folding ΔΔG of the protein upon mutation using the FoldX version 5 BuildModel command with default parameters (Delgado et al., 2019, 2025; Schymkowitz et al., 2005). Although exposed‐NE mutations are less likely to affect the stability of the protein, aromatic‐arginine stacking interactions in the protein can contribute significantly to folding stability (Delgado et al., 2025) (Table S1); thus, we calculated the folding ΔΔG for both buried‐NE and exposed‐NE mutations. A positive folding ΔΔG indicates that protein folding is destabilized (the protein falls apart), while a negative folding ΔΔG indicates that protein folding is stabilized. If a buried‐NE or exposed‐NE mutation causes the ΔΔG of the protein to be ≥2 kcal/mol, we predicted that the mutation disrupts the protein's stability and deemed it “quasi‐null” (Ghadie & Xia, 2021; Sahni et al., 2015). On the contrary, if a buried‐NE or exposed‐NE mutation causes the folding ΔΔG to be <2 kcal/mol, we predicted that the mutation maintains the stability of the protein and categorized it as “quasi‐wildtype.”

### 5.6 Constructing and mapping missense and nonsense mutations onto the human SP

To acquire supersets of missense and nonsense mutations, we constructed a human SP consisting of 3D structures for proteins in the UniProtKB/Swiss‐Prot (The UniProt Consortium, 2023) human reference proteome. We aligned the sequences of the Swiss‐Prot proteins in the proteome against all the PDB chain sequences in PDB SEQRES (Berman et al., 2000) using BLASTP (Camacho et al., 2009). For each Swiss‐Prot protein, we selected the best homologous PDB chain sequence by first filtering for ones that met the PDB resolution threshold of ≤3.5 Å and the BLASTP alignment bitscore of ≥50. If more than one PDB chain met both thresholds and had the same BLASTP alignment bitscore, the first one was selected and deemed the structural template for that Swiss‐Prot protein. We inputted the BLASTP alignments of the Swiss‐Prot proteins against their structural templates into MODELLER to construct 3D structural models. The resulting 3D structural models of the proteins in the UniProtKB/Swiss‐Prot human reference proteome made up our SP.

We processed dbSNP non‐pathogenic and ClinVar Mendelian disease‐causing missense and nonsense mutations as stated in the “Processing Mendelian disease‐causing and non‐pathogenic mutations” subsection and mapped them onto our SP as detailed in the “Mapping mutations onto human SIs” subsection. If the position of a mutation in a Swiss‐Prot protein was not found in the constructed 3D structural model for the protein, we assumed that the mutation position was not structurally resolved and discarded the mutation. We termed the dbSNP and ClinVar missense and nonsense mutations that mapped onto our SP as our supersets of missense and nonsense mutations, respectively, as more mutations are obtained when PPIs are not considered. For each of our dbSNP and ClinVar missense supersets, we identified mutations buried in the core of the protein by selecting those with RSA ≤0.25 (Ghadie & Xia, 2021; Gong et al., 2017; Xu et al., 2012), as calculated through a protocol that employs the mkDSSP software (Kabsch & Sander, 1983; Sydykova et al., 2018). We further determined missense mutations affecting the stability of the protein by selecting those with folding ΔΔG ≥2 kcal/mol (Ghadie & Xia, 2021), as computed by the FoldX version 5 BuildModel command with default parameters (Delgado et al., 2019, 2025; Schymkowitz et al., 2005).

#### CODE AVAILABILITY

The scripts written to conduct this study are publicly available on GitHub and FigShare at https://github.com/tingyisu/MutDeleteriousness and https://doi.org/10.6084/m9.figshare.27733923.v2.

## AUTHOR CONTRIBUTIONS

**Ting‐Yi Su:** Writing – original draft. **Yu Xia:** Writing – review and editing.

## FUNDING INFORMATION

This work was supported by grants from the Natural Sciences and Engineering Research Council of Canada (RGPIN‐2019‐05952 to Y.X., RGPAS‐2019‐00012 to Y.X.), the Canada Foundation for Innovation (JELF‐33732 to Y.X., IF‐33122 to Y.X.), and the Canada Research Chairs program (CRC‐2022‐00424 to Y.X.). T.‐Y.S. is supported by a doctoral fellowship from the Fonds de recherche du Québec–Nature et technologies (FRQNT).

## Supporting information


**Data S1.** Supporting Information.

## Data Availability

Data sharing is not applicable to this article as no new data were created or analyzed in this study.
