# Supplementary material for: A quantitative comparison of the deleteriousness of missense and nonsense mutations using the structurally resolved human protein interactome
Source: Protein Sci. 2025 May 19;34(6):e70155. doi: 10.1002/pro.70155 (PMC12086521; doi:10.1002/pro.70155)
Supplement: Supplementary file 1 — Data S1. Supporting Information. [file PRO-34-e70155-s001.pdf]

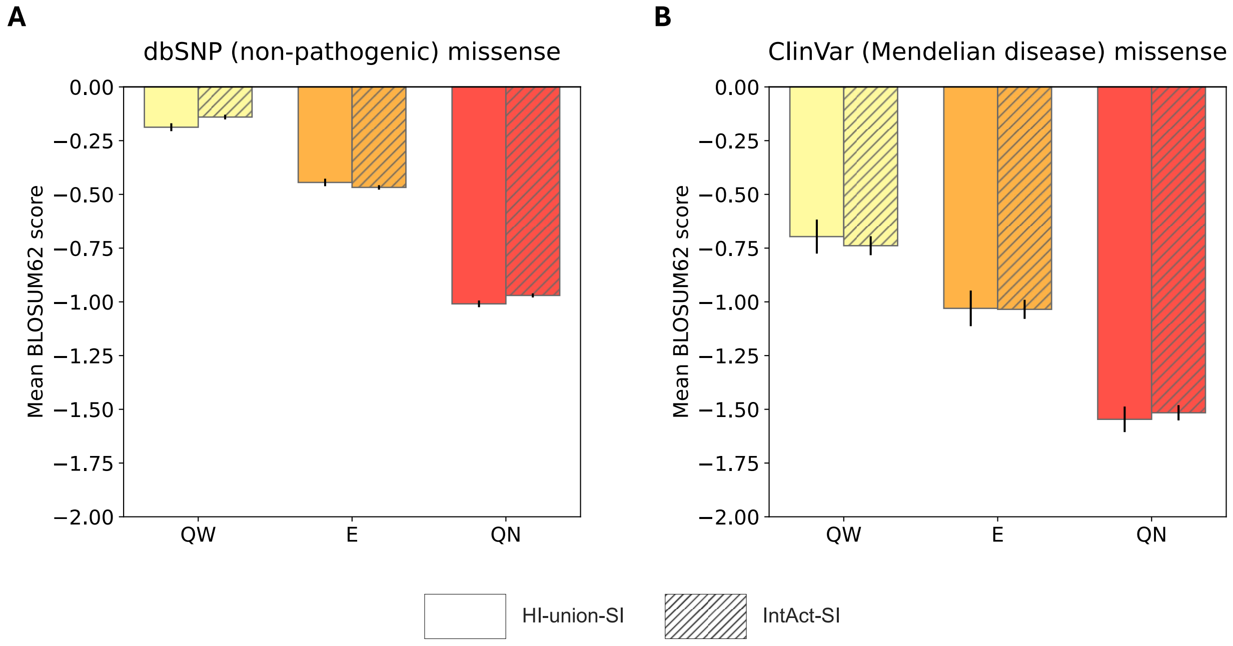

Figure S1. Investigating the conservation of amino acid substitutions in missense mutations. Mean BLOSUM62 (Henikoff & Henikoff, 1992) scores of quasi-wildtype (QW, yellow), edgetic (E, orange), and quasi-null (QN, bright red) mutations within datasets of **(A)** dbSNP non-pathogenic and **(B)** ClinVar Mendelian disease-causing missense mutations affecting proteins in the HI-union-SI (unhatched bars) and IntAct-SI (hatched bars).

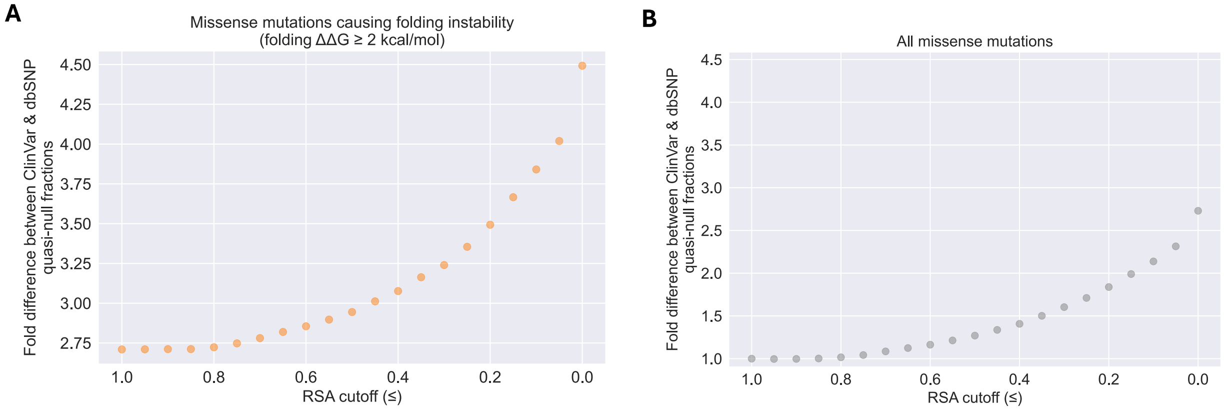

Figure S2. Investigating the connection between the fold differences (between ClinVar and dbSNP quasi-null fractions) and the relative solvent accessibility (RSA) values of the mutations in the structural proteome. Plot displaying the quadratic/exponential correlation between fold differences and RSA cutoffs ranging from 1.0 (mutation is completely exposed) to 0.0 (mutation is completely buried), for two possible alternative definitions of quasi-null mutations: **(A)** missense mutations that induce folding instability (folding $\Delta\Delta G \geq 2$ kcal/mol) and satisfy a particular RSA threshold and **(B)** all missense mutations that meet a certain RSA cutoff, without considering folding $\Delta\Delta G$ values.

A

Dimer (PPI)	MODELLER (M) dimer model (template: % identity)	AlphaFold3 (AF3) dimer model (pTM, ipTM)	RMSD (Å) between M & AF3 dimer models	RMSD (Å) between the 1st monomers in M & AF3 dimer models	RMSD (Å) between the 2nd monomers in M & AF3 dimer models	# of missense mutations in dimer
P48165<br>P29033	7jm9_L: 96.4%<br>7jm9_G: 56.0%	pTM = 0.56<br>ipTM = 0.69	0.8	0.9	0.8	10 ClinVar
P09105<br>P68871	6kye_K: 61.4%<br>6kye_L: 100.0%	pTM = 0.89<br>ipTM = 0.88	4.5	0.8	0.5	7 ClinVar
P60709<br>P40123	6rsw_A: 93.8%<br>6rsw_C: 66.3%	pTM = 0.88<br>ipTM = 0.87	2.1	0.8	0.4	6 ClinVar<br>5 dbSNP
Q92963<br>Q3MIN7	5cm8_B: 44.4%<br>5cm8_A: 44.6%	pTM = 0.83<br>ipTM = 0.88	3.6	1.3	0.8	3 ClinVar
Q9UMW8<br>P05161	5chv_A: 76.9%<br>5chv_C: 65.4%	pTM = 0.91<br>ipTM = 0.90	2.6	0.5	2.5	6 dbSNP
P23297<br>P33764	3nso_B: 43.2%<br>3nso_A: 100.0%	pTM = 0.71<br>ipTM = 0.68	3.5	1.2	0.7	6 dbSNP
Q09470<br>Q16322	7ssx_B: 89.9%<br>7ssx_A: 79.8%	pTM = 0.67<br>ipTM = 0.67	4.1	5.3	2.1	11 dbSNP
P62877<br>Q92990	4f52_D: 100.0%<br>4f52_F: 100.0%	pTM = 0.79<br>ipTM = 0.68	1.7	0.5	1.7	13 dbSNP
Q9NRX1<br>Q9ULX3	7wu0_x: 100.0%<br>7wu0_y: 100.0%	pTM = 0.39<br>ipTM = 0.40	10.3	1.0	7.5	23 dbSNP
Q9UI10<br>P49770	7kmf_E: 100.0%<br>7kmf_D: 100.0%	pTM = 0.74<br>ipTM = 0.85	3.6	0.8	0.7	10 dbSNP

MODELLER metrics

- **% identity**: sequence identity between structural template (PDB chain) and protein sequence (excluding gaps)

AlphaFold3 metrics

- **pTM** (predicted template modeling score): > 0.5 means overall predicted complex structure might be similar to the true structure
- **ipTM** (interface predicted template modeling score): accuracy of predicted relative positions of the subunits in the complex
  - > 0.8 means confident high-quality prediction
  - 0.6 – 0.8 means gray zone (prediction could be correct/incorrect)
  - < 0.6 means likely a failed prediction

B

# of dimers	# of missense mutations in dimers	Total # of missense mutations with edgotypes changed from M to AF3 dimer models			
		E→NE	QN→QW	QW→QN	Total
10	26 ClinVar, 74 dbSNP	4 (4%)	1 (1%)	6 (6%)	11 (11%)

Figure S3. Comparing the structural and mutation edgotype predictions between MODELLER and AlphaFold3 models of 10 randomly selected dimers. 10 dimers (consisting of 20 unique monomers) present in both our HI-union-SI and IntAct-SI and comprising mutations across all 3 edgotypes (quasi-wildtype, edgetic, and quasi-null) were randomly selected for AlphaFold3 (Abramson et al., 2024) dimer structure predictions. **(A)** Table illustrating the MODELLER and AlphaFold3 prediction metrics of the 10 dimers modelled, the RMSDs (Å) between the 10 dimers (and their corresponding 20 monomers) modelled by MODELLER and their respective counterparts predicted by AlphaFold3, and the number of ClinVar and dbSNP mutations in each dimer. **(B)** Table comparing the differences in the predicted edgotypes of missense

mutations in MODELLER versus AlphaFold3-constructed structural PPI models. ‘E→NE’ represents a missense mutation that disrupted the corresponding interaction (binding $\Delta\Delta G > 0.8$ kcal/mol) in the MODELLER model but not (binding $\Delta\Delta G \leq 0.8$ kcal/mol) in the AlphaFold3 model. ‘QN→QW’ represents a missense mutation that disrupted the stability of the corresponding monomer (folding $\Delta\Delta G \geq 2$ kcal/mol) in the MODELLER model but not (folding $\Delta\Delta G < 2$ kcal/mol) in the AlphaFold3 model. ‘QW→QN’ represents a missense mutation that did not disrupt the stability of the corresponding monomer (folding $\Delta\Delta G < 2$ kcal/mol) in the MODELLER model but did (folding $\Delta\Delta G \geq 2$ kcal/mol) in the AlphaFold3 model.

	dbSNP		ClinVar	
	Exposed-NE		Exposed-NE	
	< 2 kcal/mol	≥ 2 kcal/mol (*)	< 2 kcal/mol	≥ 2 kcal/mol (*)
HI-union-SI	**4,553**	**283 (233)**	**143**	**11 (7)**
IntAct-SI	**12,534**	**754 (653)**	**383**	**38 (26)**

* = # with folding ΔΔG ≥ 2 kcal/mol and have aromatic-arginine stacking interactions in the monomer

Table S1. Table depicting the number of exposed-NE mutations that affect folding stability due to aromatic-arginine stacking interactions. Both parallel and T-stacking aromatic-arginine interactions were considered. Exposed-NE mutations are defined as missense mutations that are exposed on the surface of the protein and do not affect the protein's interactions (non-edgetic). The number of exposed-NE mutations with folding ΔΔG ≥ 2 kcal/mol that also have aromatic-arginine stacking interactions in the protein is shown in parentheses and represented by a *.

A

% of missense mutations that are quasi-null		
	**dbSNP**	**ClinVar**
First 5% of protein	4.5%<br>3.6%	0.0%<br>11.8%
Last 5% of protein	6.5%<br>5.3%	17.6%<br>20.5%
Whole protein	6.5%<br>7.5%	19.3%<br>25.2%

HI-union-SI IntAct-SI

B

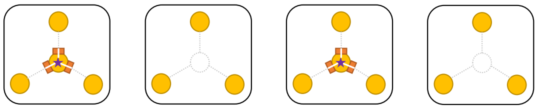

Distribution of quasi-null and nonsense mutations				
	**dbSNP**		**ClinVar**	
	quasi-null	nonsense	quasi-null	nonsense
First 5% of protein	14 (2.6%)<br>30 (1.7%)	6 (3.6%)<br>16 (3.8%)	0 (0.0%)<br>8 (2.3%)	12 (4.3%)<br>56 (3.9%)
Last 5% of protein	19 (3.5%)<br>53 (3.0%)	7 (4.2%)<br>17 (4.0%)	3 (3.5%)<br>9 (2.6%)	4 (1.4%)<br>26 (1.8%)

Figure S4. Investigating the distribution of quasi-null and nonsense mutations in the first 5% and last 5% of proteins in the HI-union-SI (blue) and IntAct-SI (green). (A) Table showing the numbers and percentages of dbSNP and ClinVar missense mutations in the first 5%, last 5%, and entire protein that result in quasi-null mutations. **(B)** Table displaying the numbers and percentages of dbSNP and ClinVar quasi-null and nonsense mutations distributed in the first 5% and last 5% of proteins.

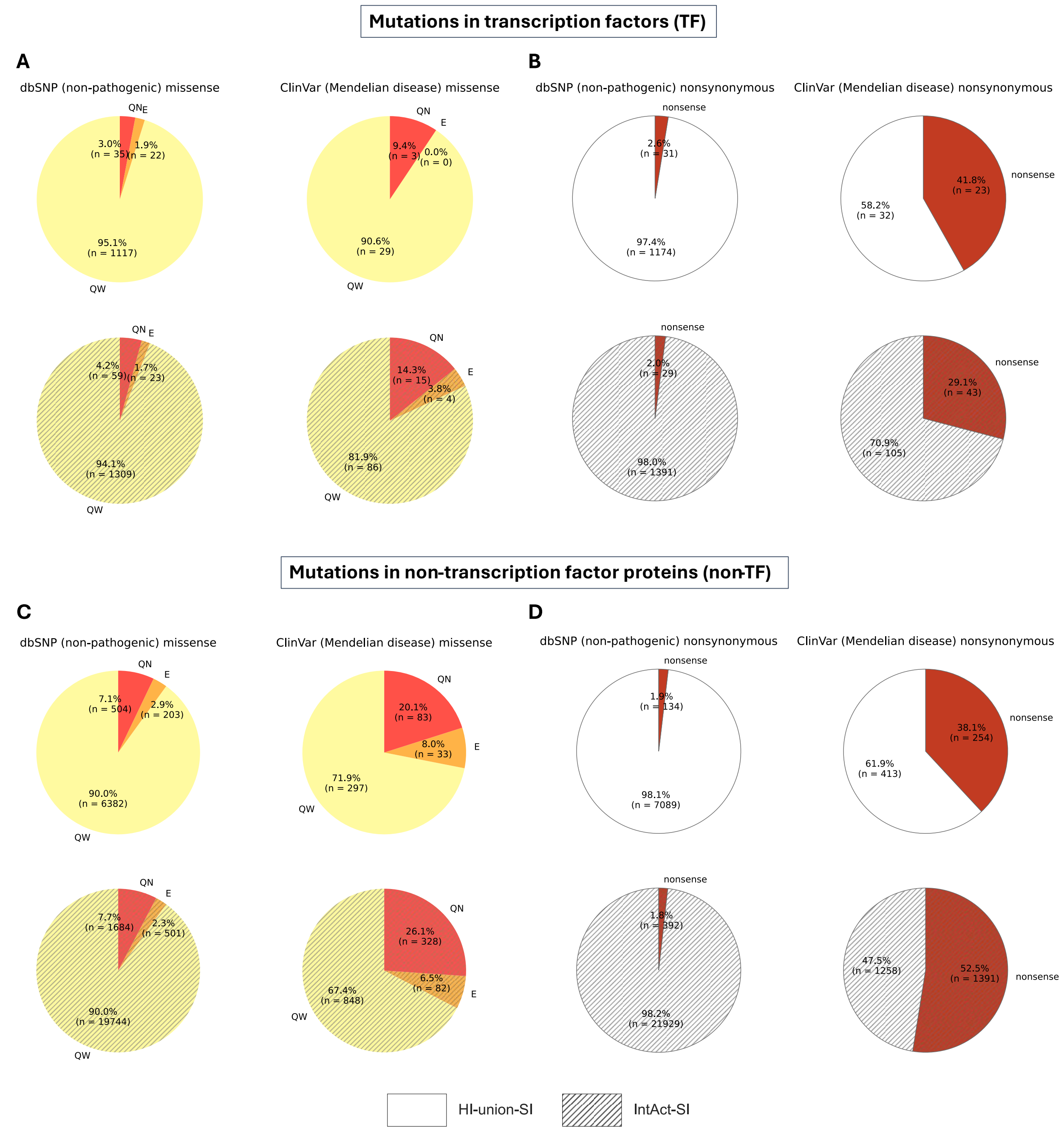

Figure S5. Investigating the edgotypes of missense mutations and enrichment of nonsense mutations in transcription factor (TF) and non-transcription factor (non-TF) proteins in the HI-union-SI (hatched) and IntAct-SI (unhatched). TF proteins were obtained using the 'DNA-binding transcription factor activity (GO:0003700)' annotation on UniProtKB (The UniProt Consortium, 2023). Edgotypes of missense mutations include quasi-wildtype (QW, yellow), edgetic (E, orange), and quasi-null (QN, bright red) mutations. Nonsense mutations are shown in dark red. Piecharts displaying the **(A)** edgotypes of missense mutations and **(B)** enrichment of nonsense mutations in TFs, as well as **(C)** edgotypes of missense mutations and **(D)** enrichment of nonsense mutations in non-TFs.

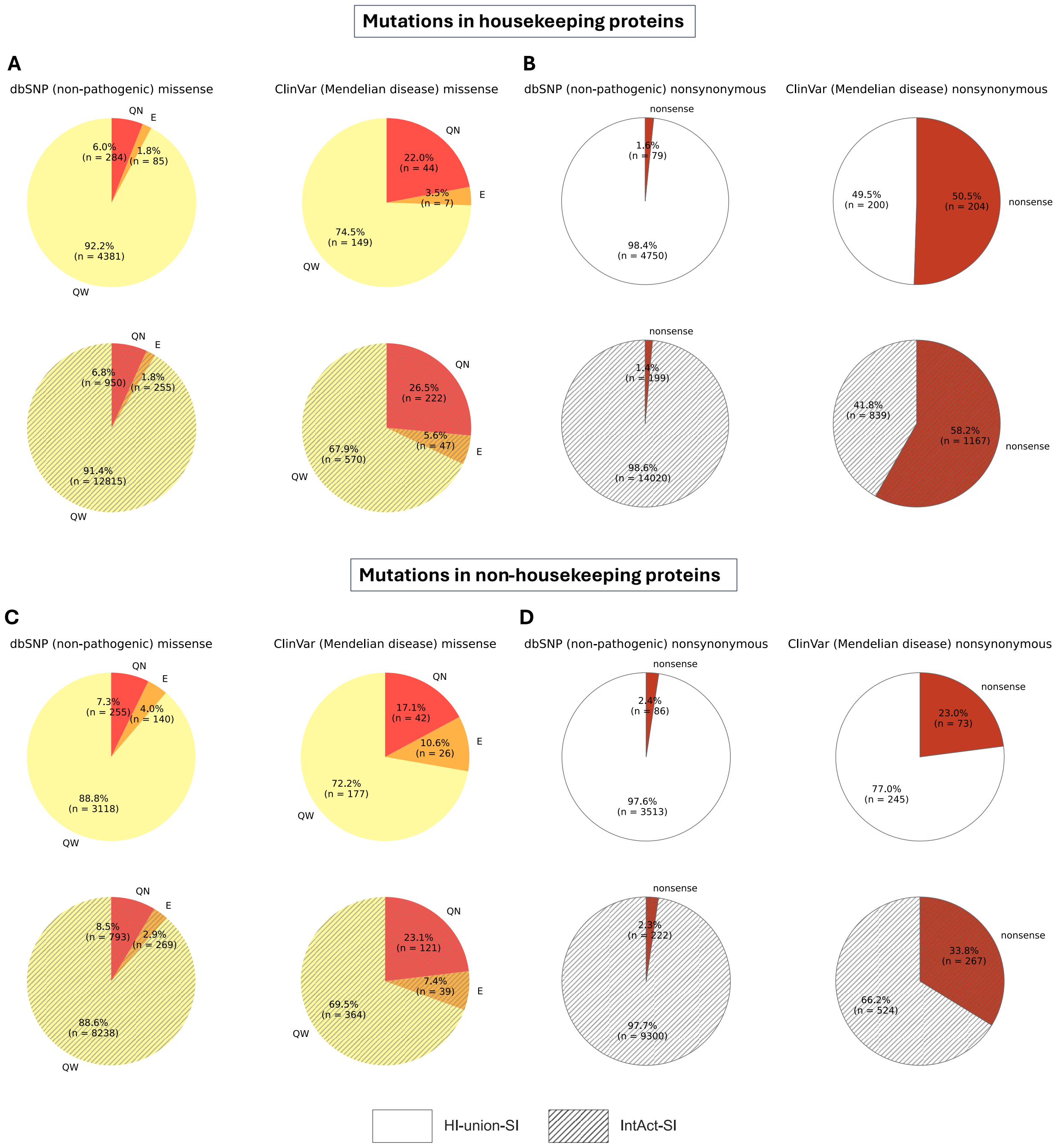

Figure S6. Investigating the edgotypes of missense mutations and enrichment of nonsense mutations in housekeeping and non-housekeeping proteins in the HI-union-SI (hatched) and IntAct-SI (unhatched). Housekeeping proteins were obtained from the Human Protein Atlas (Uhlén et al., 2015). Edgotypes of missense mutations include quasi-wildtype (QW, yellow), edgetic (E, orange), and quasi-null (QN, bright red) mutations. Nonsense mutations are shown in dark red. Piecharts displaying the **(A)** edgotypes of missense mutations and **(B)** enrichment of nonsense mutations in housekeeping proteins, as well as **(C)** edgotypes of missense mutations and **(D)** enrichment of nonsense mutations in non-housekeeping proteins.

A

		HI-union-SI	IntAct-SI	Overall
**Transcription factor (TF)**	Quasi-null	3.13	3.40	**3**
	Nonsense	16.08	14.55	**15**
**Non-transcription factor (non-TF)**	Quasi-null	2.83	3.39	**3**
	Nonsense	20.05	29.17	**20**

B

		HI-union-SI	IntAct-SI	Overall
**Housekeeping protein**	Quasi-null	3.67	3.90	**>3**
	Nonsense	31.56	41.57	**30**
**Non-housekeeping protein**	Quasi-null	2.34	2.72	**<3**
	Nonsense	9.58	14.70	**10**

Figure S7. Investigating the fold differences of quasi-null and nonsense mutations in proteins with different functional categories across the HI-union-SI and IntAct-SI. Fold differences (between ClinVar and dbSNP quasi-null or nonsense fractions shown in Figures S5 and S6) for node-removal mutations in **(A)** transcription factor and non-transcription proteins and **(B)** housekeeping and non-housekeeping proteins. The 'Overall' column shows the estimated fold differences derived from the HI-union-SI and IntAct-SI.

A

Mutations mapped onto structural proteome (SP)				
	**dbSNP**		**ClinVar**	
	Transcription factor (TF)	Non-transcription factor (non-TF)	Transcription factor (TF)	Non-transcription factor (non-TF)
# of nonsense mutations	227	2,528	112	3,929
# of missense mutations	6,135	108,522	209	4,083

Mutations in transcription factors (TF)

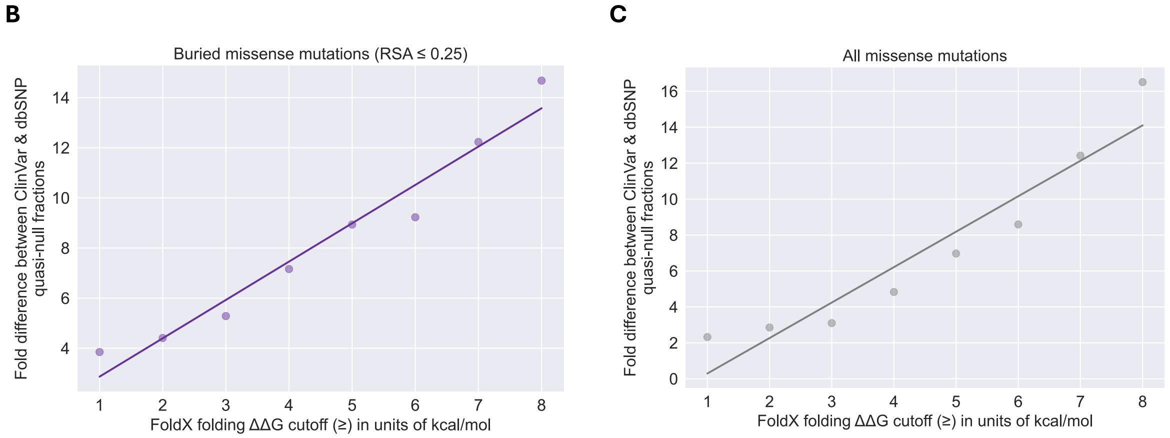

Mutations in non-transcription factor proteins (non-TF)

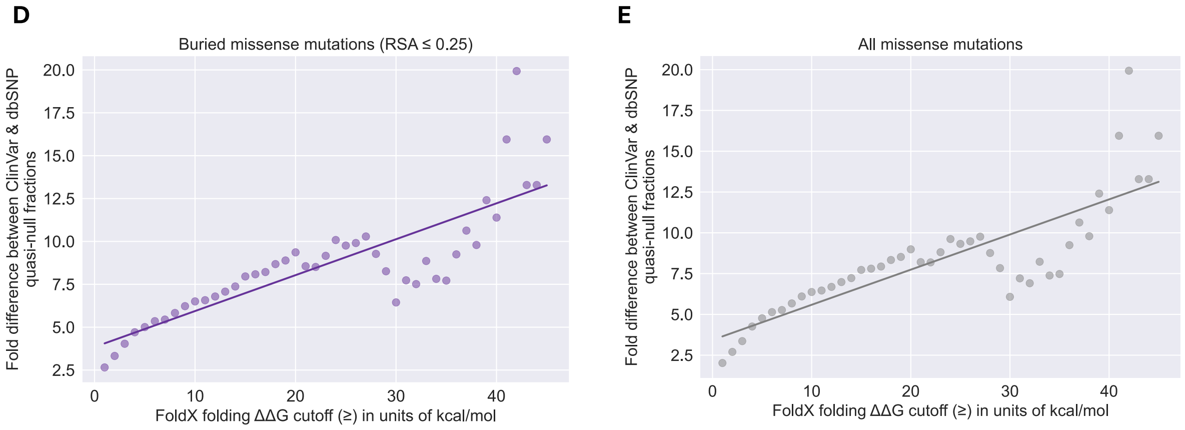

Figure S8. Assessing the relationship between the fold difference and folding instability of quasi-null mutations mapped onto transcription factor (TF) and non-transcription factor (non-TF) proteins in the structural proteome. (A) Table depicting the numbers of dbSNP and ClinVar nonsense and missense mutations in TF and non-TF proteins. Plots portraying the relationship between the fold differences and folding ΔΔG cutoffs (1 to 8 kcal/mol for TFs and 1 to 45 kcal/mol for non-TFs) for quasi-null mutations in TFs and non-TFs, using two different definitions of quasi-null mutations in which the level of burial of the mutations (RSA ≤ 0.25) is either considered **(B, D)** or not **(C, E)**.

A

Mutations mapped onto structural proteome (SP)				
	dbSNP		ClinVar	
	Housekeeping	Non-housekeeping	Housekeeping	Non-housekeeping
# of nonsense mutations	764	1,991	2,237	1,804
# of missense mutations	43,852	70,805	1,812	2,480

Mutations in housekeeping proteins

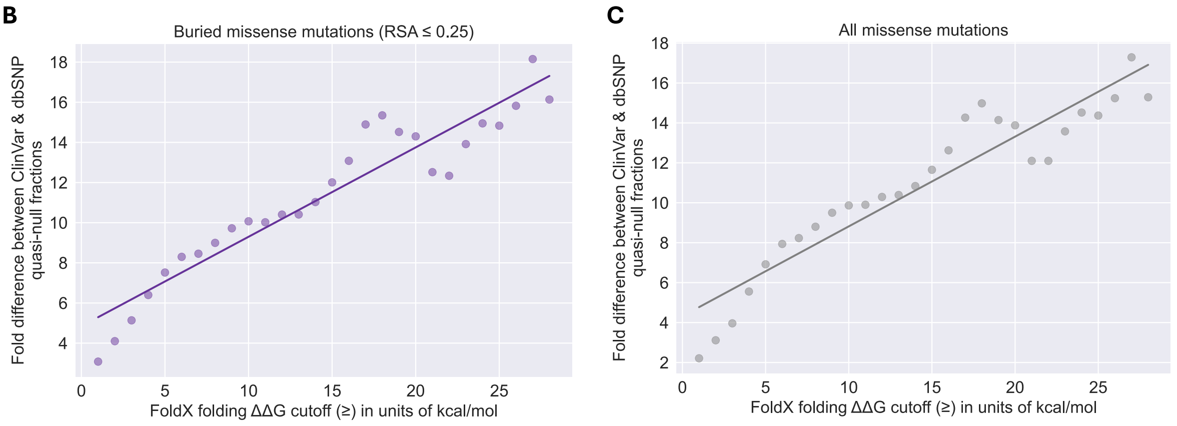

Mutations in non-housekeeping proteins

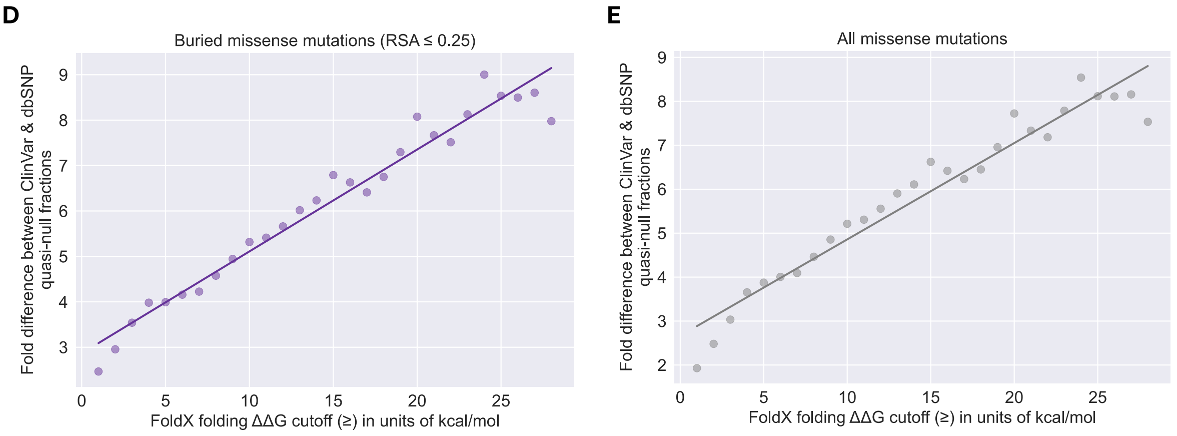

Figure S9. Assessing the relationship between the fold difference and folding instability of quasi-null mutations mapped onto housekeeping and non-housekeeping proteins in the structural proteome. (A) Table depicting the numbers of dbSNP and ClinVar nonsense and missense mutations in housekeeping and non-housekeeping proteins. Plots portraying the relationship between the fold differences and folding ΔΔG cutoffs (1 to 28 kcal/mol) for quasi-null mutations in housekeeping and non-housekeeping proteins, using two different definitions of quasi-null mutations in which the level of burial of the mutations (RSA ≤ 0.25) is either considered **(B, D)** or not **(C, E)**.

A **Temporal nature of PPIs**

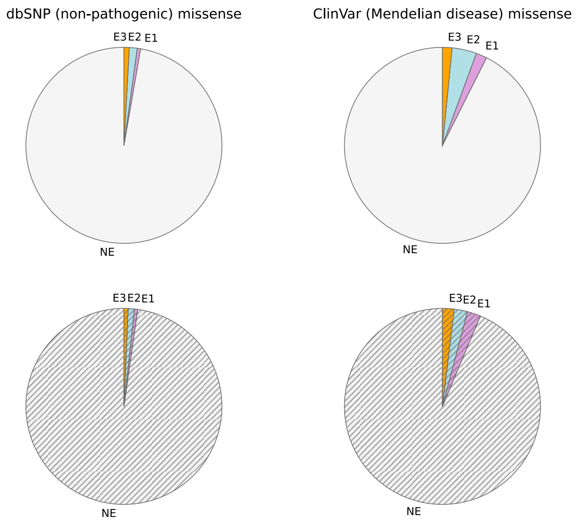

NE (gray) = non-edgetic (quasi-wildtype & quasi-null)
E1 (purple) = disrupts transient PPIs
E2 (blue) = disrupts permanent PPIs
E3 (orange) = other (neither/both E1 and E2)

	**dbSNP**			**ClinVar**		
	E1	E2	E3	E1	E2	E3
HI-union-SI	45 (0.5%)	107 (1.3%)	73 (0.9%)	8 (1.8%)	18 (4.0%)	7 (1.6%)
IntAct-SI	123 (0.5%)	240 (1.0%)	161 (0.7%)	30 (2.2%)	30 (2.2%)	26 (1.9%)

B **Tissue-specific nature of PPIs**

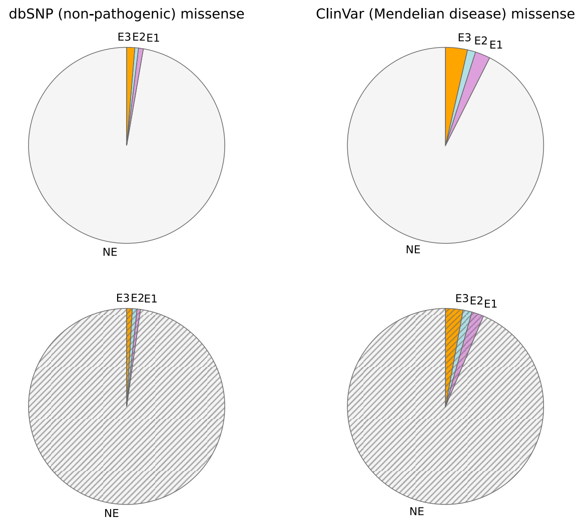

NE (gray) = non-edgetic (quasi-wildtype & quasi-null)
E1 (purple) = disrupts tissue-specific PPIs
E2 (blue) = disrupts non-tissue-specific PPIs
E3 (orange) = other (neither/both E1 and E2)

	**dbSNP**			**ClinVar**		
	E1	E2	E3	E1	E2	E3
HI-union-SI	66 (0.8%)	49 (0.6%)	110 (1.3%)	11 (2.5%)	6 (1.3%)	16 (3.6%)
IntAct-SI	131 (0.5%)	179 (0.8%)	214 (0.9%)	27 (2.0%)	20 (1.4%)	39 (2.9%)

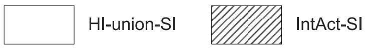

Figure S10. Distinguishing edgetic mutations based on the temporal nature or tissue-specific nature of the protein-protein interactions (PPIs) disrupted in the HI-union-SI (hatched) and IntAct-SI (unhatched). Two types of PPI categorizations were investigated: transient versus permanent, and tissue-specific versus non-tissue-specific. PPI categorizations were obtained from a recent study (Ghadie & Xia, 2022) using human gene expression data across different time courses (Barrett et al., 2013) and tissues (Illumina, Inc., 2011). Piecharts and table depicting the fractions of missense mutations that **(A)** disrupt only transient PPIs (E1), only permanent PPIs (E2), or PPIs that are not clearly defined as either transient or permanent (E3), as well as **(B)** disrupt only tissue-specific PPIs (E1), only non-tissue-specific PPIs (E2), or PPIs that are not clearly defined as either tissue-specific or non-tissue-specific (E3).

References

Abramson, J., Adler, J., Dunger, J., Evans, R., Green, T., Pritzel, A., … Jumper, J. M. (2024). Accurate structure prediction of biomolecular interactions with AlphaFold 3. *Nature*, *630*(8016), 493–500. https://doi.org/10.1038/s41586-024-07487-w

Barrett, T., Wilhite, S. E., Ledoux, P., Evangelista, C., Kim, I. F., Tomashevsky, M., … Soboleva, A. (2013). NCBI GEO: Archive for functional genomics data sets—update. *Nucleic Acids Research*, *41*(D1), D991–D995. https://doi.org/10.1093/nar/gks1193

Ghadie, M., & Xia, Y. (2022). Are transient protein-protein interactions more dispensable? *PLOS Computational Biology*, *18*(4), e1010013. https://doi.org/10.1371/journal.pcbi.1010013

Henikoff, S., & Henikoff, J. G. (1992). Amino acid substitution matrices from protein blocks. *Proceedings of the National Academy of Sciences*, *89*(22), 10915–10919. https://doi.org/10.1073/pnas.89.22.10915

Illumina, Inc. (2011). *Illumina Human Body Map 2.0 Project* [Data set]. https://www.ncbi.nlm.nih.gov/geo/query/acc.cgi?acc=GSE30611: NCBI Gene Expression Omnibus (GEO).

The UniProt Consortium. (2023). UniProt: The Universal Protein Knowledgebase in 2023. *Nucleic Acids Research*, *51*(D1), D523–D531. https://doi.org/10.1093/nar/gkac1052

Uhlén, M., Fagerberg, L., Hallström, B. M., Lindskog, C., Oksvold, P., Mardinoglu, A., … Pontén, F. (2015). Tissue-based map of the human proteome. *Science*, *347*(6220), 1260419. https://doi.org/10.1126/science.1260419
